# Long-term chronicity of work addiction: the role of personality and work motivations

**DOI:** 10.1186/s40359-025-02907-6

**Published:** 2025-05-29

**Authors:** Bernadette Kun, Gyöngyi Kökönyei

**Affiliations:** 1 Institute of Psychology, ELTE Eötvös Loránd University, Budapest, Hungary; 2 NAP3.0 - SE Neuropsychopharmacology Research Group, Hungarian Brain Research Program, Semmelweis University, Budapest, Hungary; 3 Department of Pharmacodynamics, Faculty of Pharmaceutical Sciences, Semmelweis University, Budapest, Hungary

**Keywords:** Work addiction, Workaholism, Personality, Work motivation, Psychopathological distress, Longitudinal

## Abstract

The current comprehensive study investigated the relationship between work addiction (WA), personality traits, and motivations, focusing on their roles in WA persistence and recovery. The objective was to explore the capacity of personality dimensions and work motivation to predict the chronicity of WA, symptom variation, and gender differences. Over a four-year study of 586 participants (51.4% female), the Bergen Work Addiction Scale was used to assess WA at two time points. Individual differences were assessed by Rosenberg Self-Esteem Scale, Narcissistic Admiration and Rivalry Questionnaire, Multidimensional Perfectionism Scale, short Brief Symptom Inventory, Ruminative Response Scale, and Multidimensional Work Motivation Scale at Time 1. Latent class analysis identified two primary groups in the entire sample: individuals with persistent WA and those without. While females aligned with these groups, males exhibited five distinct latent classes. In females, chronic WA was associated with lower self-esteem, higher self-oriented and socially prescribed perfectionism, rumination, psychological distress, and increased extrinsic motivation. In males, the chronic WA group exhibited heightened levels of perfectionism, rumination, psychopathological distress, lower self-esteem, and elevated extrinsic and introjected regulations compared to the other WA groups. These findings emphasize the possibility of WA persisting over an extended period, with personality traits and motivations playing a significant role in this endurance. These results not only advance our understanding of WA but also provide valuable insights for future research on protective factors against WA. Furthermore, the findings have significant practical implications, including addressing excessive perfectionism in work environments and offering avenues for future research.

**Supplementary Information:**

The online version contains supplementary material available at 10.1186/s40359-025-02907-6.

## Introduction

Determining the underlying factors of a mental health issue poses a significant challenge. This holds true when investigating work addiction, as unraveling the precursors of this disorder is a daunting task. Numerous theories and studies have identified various macro-level, meso-level, and micro-level factors of work addiction [1] but most of these findings based on cross-sectional studies [2]. Although an increasing amount of longitudinal research is emerging, there still exist significant gaps in understanding the individual factors contributing to changes in severity, particularly regarding the long-term persistence of work addiction. Are there specific individual factors, such as personality traits and motivational aspects, that predict changes in work addiction over time, including its stability or potential symptom reduction, as well as membership in symptom-based groups or distinct trajectories of work addiction? The current study aims to address these questions and fill this research gap.

### Work addiction

The field of work addiction research has experienced a significant surge in recent decades, primarily due to its high prevalence, estimated to between 7% and 39% [3–6]. Nevertheless, substantial debate persists regarding the phenomenon, leading to varying perspectives on its definition. Among the various definitions proposed, we present the one employed by Atroszko et al. (7 p. 9), which delineates work addiction as “*characterized by a compulsion to work and preoccupation with work activities leading to a significant harm and distress of a functionally impairing nature to the individual and/or other significantly relevant relationships (friends and family). The behavior is characterized by the loss of control over the working activity and persists over a significant period of time*.” This definition underscores the close resemblance of work addiction to other addictive disorders and highlights the likely contribution of internal characteristics (e.g., compulsivity, loss of control) in the onset of symptoms. Based on numerous studies, work addiction is associated with several adverse factors that affect a person’s physical and mental health, social relationships, and even career. Individuals with work addiction report higher levels of stress and anxiety [8, 9], more depressive symptoms [10], more physical ailments [11, 12], consume more stimulant drugs [13], and characterized by more risky mobile phone use [14]. They are more likely to experience conflicts between work and family [15–17], express dissatisfaction in their romantic relationships [18], encounter conflicts with the supervisors [19], have a higher prevalence of burnout [20, 21], and show less satisfaction with job [22].

This multitude of unfavorable characteristics not only highlights that work addiction is a harmful phenomenon but also draws attention to the importance of understanding its causes and persistence. While macro-level factors (e.g., society, culture, economy) and meso-level factors (e.g., organizational culture and climate, workplace requirements and reward systems) undoubtedly contribute to the development of work addiction [23, 24], the significance of individual factors cannot be overlooked. Despite individuals residing in the same country, sharing a common culture, or working within the same organization, not everyone will exhibit work addiction tendencies. This underscores the long-term effects of individual factors, such as personality traits and motivational factors, on work addiction.

### Work addiction and personality

Research on work addiction has traditionally placed significant emphasis on the role of personality traits. This emphasis can be traced back to the early definitions of work addiction proposed by Oates [25] and Robinson [26], which highlighted the importance of certain traits, such as compulsiveness and perfectionism, in the symptoms of the disorder. It is noteworthy that personality traits also play an important role in the etiology of both substance-related and behavioral addictions [27, 28]. Two comprehensive meta-analyses have been conducted in the field of work addiction that addressed the role of individual factors: one focused on the subject of work addiction itself, while the other specifically examined its relationship with personality. The findings of Clark et al.’s [2] study revealed positive associations between work addiction and extraversion, negative affectivity, perfectionism, and Type A personality. Building on this, a subsequent meta-analysis conducted by Kun and colleagues [29] identified four personality dimensions that exhibited the most substantial relationship with work addiction. Specifically, negative affectivity, perfectionism, and performance-based self-esteem demonstrated positive associations with work addiction, whereas global self-esteem displayed a negative association. It is important to note that while there are other personality traits significantly associated with work addiction, the body of research on these traits is relatively limited, with only one or two studies conducted thus far. These additional traits include compulsiveness, trait anxiety, Type A personality, narcissism, and persistence [29].

These two meta-analyses have also highlighted the paucity of longitudinal research analyzing the role of personality traits in work addiction. To the best of our knowledge, only three such longitudinal studies have been conducted, with two of them primarily focusing on assessing the Big Five traits [30–32], although the more recent meta-analysis [29] established that the impact of these traits on work addiction is negligible in cross-sectional designs. In a longitudinal study involving workers from two European countries, Atroszko et al. [31] examined the predictive value of the Big Five factors at time 1 (T1) on work addiction at time 2 (T2). The results indicated that among the T1 Big Five factors, only neuroticism demonstrated a significant predictive relationship with T2 work addiction. Notably, this association was observed solely within the Norwegian sample, with none of the traits yielding significant predictions among the Polish participants. Furthermore, another study [30] conducted specifically among Norwegian nurses employed the Bergen Work Addiction Scale to categorize participants into four distinct groups based on the cut-off point. These groups consisted of individuals who were not affected by work addiction at either time point, those who exhibited work addiction tendencies at both measurement time, those who displayed an increase in work addiction symptoms over time, and those who exhibited a decrease in symptoms over time. They found that stable non-addicted workers exhibited lower neuroticism scores during the initial data collection compared to the other three groups. Additionally, stable non-addicted workers displayed lower intellect/imagination scores at T1 compared to those who showed stable work addiction. Finally, in an Italian study with a 3-month follow-up, it was found that earlier self-oriented perfectionism was associated with higher work addiction later on, but only when workload was also higher [32].

In addition to the aforementioned considerations, the relationship between rumination and work addiction has garnered attention in recent years. Rumination can be conceptualized as both a state and a trait [33], with the latter being closely associated with personality. The trait rumination refers to a stable pattern of repetitively focusing on negative aspects of one’s life, often accompanied by heightened depressive symptoms [34]. Given that individuals with work addiction exhibit higher levels of anxiety, depressive states, and less satisfaction with themselves [35, 36], it has been hypothesized that these people may display more maladaptive rumination tendencies. Empirical studies have provided support for this proposition [37], although limited data exists regarding the extent to which maladaptive rumination can serve as a predictor of work addiction symptoms.

Personality traits are fundamental in understanding the stability or change in work addiction over time, as they represent enduring individual differences that might influence how individuals perceive, interpret, and respond to work-related demands. By definition, personality traits are relatively stable over time, reflecting consistent patterns of thought, emotion, and behavior [38]. This temporal stability suggests that traits may exert a particularly strong effect on long-term behavioral patterns, such as the persistence or remission of work addiction symptoms. Traits such as perfectionism, low self-esteem, and a tendency toward rumination can shape coping strategies and motivational orientations, which may either maintain or mitigate maladaptive work behaviors. Thus, examining personality traits provides a crucial framework for understanding individual variability in the course of work addiction.

### Motivations in work addiction

When examining individual factors, it is crucial to focus on motivational factors, specifically how work motivation differs between individuals with work addiction symptoms and those without. Exploring the motives can bring us closer to understanding why someone engages in an activity compulsively, despite its harmful effects on themselves and causing significant distress. This holds true for other addictions, such as substance abuse, gambling, or gaming disorder [39–41]. Merely looking at the number of hours worked does not provide insights into addictive behavior, despite earlier studies equating work addiction with excessive work [42]. It is now evident that work addiction has only a low or moderate correlation with the number of hours worked [43, 44]. In other words, while all people affected by work addiction work extensively, not everyone who works a lot has a work addiction. Numerous external factors can contribute to overworking, unrelated to compulsiveness. For instance, individuals facing financial difficulties may need to work long hours to cover bills or mortgage payments, which is not driven by internal compulsions. Furthermore, research findings indicate a weak relationship between work addiction and salary [45]. If material motives were the main driving force, a stronger correlation would be anticipated. In contrast, work addiction was initially defined as excessive work motivated by internal compulsions [25, 26, 46]. The early theory by Spence and Robbins [47] viewed work addiction as a combination of a strong urge to work (Drive), significant involvement in work-related activities (Involvement), but without deriving enjoyment from the process (Enjoyment). This suggests that the individual is driven by an internal motive, although it is not an intrinsic motivation associated with pleasure.

To date, there has been limited research specifically dedicated to exploring work motivation in individuals affected by work addiction. Existing studies have primarily utilized the Self-Determination Theory (SDT) [48, 49] to examine work motivations within this context. According to SDT, motivation exists along a continuum of self-regulation, varying based on whether behavior is driven by external factors or internal convictions and interests. *Regulation* refers to the mechanisms governing behavior, such as external rewards, internal pressure, or identification with personal values. The more internalized the regulation, the greater the autonomy and personal commitment to the activity. While closely related, *motivation* and *regulation* are not synonymous: motivation reflects the presence and type of driving force behind behavior, whereas regulation describes the mechanisms through which it operates. Extrinsic motivation, driven by external factors, resides at one end of the spectrum. Intrinsic motivation, on the other end, leads to autonomous and self-determined behavior [49]. Extrinsic motivation involves engaging in an activity to obtain rewards, recognition, or avoid negative consequences. Intrinsic motivation, in contrast, emerges when individuals perform an action for its inherent interest, excitement, or enjoyment. SDT identifies four forms of extrinsic regulation: external regulation (based on punishments or rewards), introjected regulation (linked to feelings of shame, guilt, and inadequacy when expectations are not met), identified regulation (aligned with personal values and instrumental purposes), and integrated regulation (aligned with self-values and other values) [49].

Using the SDT model, Van den Broeck et al. [50] found that compulsive work exhibited a positive, medium-strength correlation with controlled regulation, while it showed no significant association with autonomous regulation. Furthermore, controlled regulations were more robust predictors of compulsive work compared to excessive work. In addition, several studies have reported that among the controlled regulations, introjected regulation exhibits the strongest association with work addiction [44, 51, 52]. According to these studies, individuals with work addiction are primarily compelled to continue working in order to evade negative emotional states such as anxiety, shame, or guilt.

To our knowledge, only one longitudinal study analyzed the relationship between work motivations and work addiction. Taris and colleagues [53] found that work addiction led to an increase in introjected regulation and a decrease in intrinsic motivation over time. However, these motivations did not predict future work addiction six months later. It means that individuals with work addiction rely on internalized external standards of self-worth and social approval, driven by the need to demonstrate competence and avoid negative emotions. However, this type of motivation hinders the pursuit of genuine goals and compromises intrinsic motivation and growth. The study also revealed a negative impact of work addiction on intrinsic motivation, making tasks less enjoyable due to resource depletion from excessive effort [53]. Although this study primarily demonstrates the effect of work addiction on work motivation, further research is needed to test the reverse relationship. Previous research, despite relying on cross-sectional analysis, has shown that introjected and identified regulation, when used as predictor variables in a path model, have a significant effect on work addiction [52]. These authors also hypothesized that the motivations described in SDT are linked to deep-rooted individual characteristics (e.g., feelings of low self-worth and insecurity) and may reinforce workaholic tendencies. Moreover, motivations play a significant role in other addictive disorders—whether substance-related or behavioral (e.g., alcohol use, gambling, video gaming)—as they are strong predictors of later problematic use or behavior [54–56]. Given that work addiction shares many characteristics with addictive disorders (i.e., a component model [23], it is reasonable to assume that motivations may also predict the progression of the disorder over time.

### Gender differences in work addiction

Research on work addiction often involves examining gender differences, such as variations in the extent and symptoms of work addiction between sexes or differences in the prevalence of the problem within a given population. This question is relevant not only because many behavioral addictions show strong gender disparities (e.g., gaming disorder is more common among men [57], while shopping disorder is more common among women [58], but also due to the potential impact of cultural work ethics and gender-specific role expectations in different cultures and countries. These factors can influence macro, meso, and micro-level dynamics.

Research findings on gender differences in work addiction are inconsistent, and meta-analyses suggest that there may be no significant disparity in the prevalence of work addiction between men and women [2, 29]. It is plausible that variations in the relevant factors may offset each other in terms of work addiction. For example, while narcissism might be more prevalent in men [59], women might experience lower self-esteem [60], and both of these traits are associated with work addiction. Moreover, gender-specific role expectations could potentially influence how males and females respond to questionnaire items assessing work addiction. One study [61] has suggested that the inclination to conform to these role expectations might be a reason why no gender difference is evident in certain items. For instance, women are more likely than men to agree with the following statement of the Work Addiction Risk Test [62]: ‘I find myself doing two or three things at one time, such as eating lunch and writing a memo, while talking on the phone.’, however, males are more likely to consider this item to be more specific to themselves: ‘I find myself continuing to work after my co-workers have called it quits’. The findings may suggest that certain characteristics of work addiction (e.g., multitasking), are more prevalent among women, while others (e.g., competitiveness), are more common among men. Consequently, no significant gender differences seem to emerge as a result. However, there is no evidence suggesting that measurement invariance across gender has not been found when examining work addiction questionnaires. In the case of the Bergen Work Addiction Scale, the Italian adaptation showed measurement invariance across gender [63], and a comparative study of the Italian and American versions of the Multidimensional Workaholism Scale also found no gender differences [64].

It is important to note, however, that the majority of research on work addiction relies on convenience sampling, and there is very little representative sample research available in this field. Taking this into account, we observe that while in South Korea, representing the Eastern culture, significantly more men are affected by work addiction [5]; in Western cultures, both Hungarians and Germans exhibit significantly more symptoms of work addiction among women [4, 65]. On the other hand, in Norway, no significant difference was found between the two genders [3]. It is essential to consider that these studies have primarily analyzed gender differences cross-sectionally. Therefore, it becomes crucial to explore the topic longitudinally, including investigating the role of personality and motivations.

### Aims of the present study

In the present study, our aim is to examine whether key personality dimensions and work motivations, as defined by the Self-Determination Theory, predict the long-term persistence of work addiction over a four-year period. Specifically, we address the following research objectives:

1. Prediction of long-term work addiction: We investigate how personality traits and work motivations affect the persistence of work addiction over time, using psychometrically accepted cut-off points [66].

- We hypothesize that self-esteem (H1), perfectionism (H2), narcissism (H3), and rumination (H4) will predict long-term persistent work addiction.
- Additionally, while it is not a personality trait, we hypothesize that psychological distress (H5), which is closely related to neuroticism [67], will also predict long-term persistent work addiction.
- Among work motivations, we hypothesize that introjected (H6) and identified regulations (H7) will significantly predict persistent work addiction.

2. Latent class analysis: We identify subgroups of individuals based on work addiction symptoms at both the initial data collection and follow-up, distinguishing between those whose symptoms change, remain stable, or do not appear.

- H8: We hypothesize that different trajectories of work addiction will emerge over time, including groups where symptoms decrease or increase, as well as stable groups where work addiction either remains chronic or is consistently absent.

3. Gender and personality/motivation differences: We examine gender differences in personality traits and work motivations in relation to work addiction trajectories.

- Since previous findings on gender differences in work addiction have been inconsistent, we did not formulate a specific hypothesis but conducted these analyses in an exploratory manner.

The novelty of our research lies in its longitudinal approach, exploring how personality dimensions and motivations are associated with future work addiction and its long-term changes. To achieve this, we analyze potential changes in symptom severity over time while assessing the role of personality and motivation factors.

## Method

### Participants

At T1, 4,340 participants filled out the questionnaires (female = 2,202 [50.7%], male = 2,138 [49.3%]), ranging in age from 18 to 82 years (*M* = 37.4 years, *SD* = 9.9). The sample was drawn using a convenience sampling method. Within the T1 sample of 4,340 individuals, a total of 1,743 people (40.16%) provided their email addresses for future contact during T2. Subsequently, we reached out to these individuals and obtained responses from 586 people (33.62% of those who provided their contact information) in T2. As all participants successfully completed the concise 10-minute questionnaire, data from all T2 participants were included in our analysis. Consequently, the final sample of this study consisted of 586 participants (female = 301 [51.4%], male = 285 [48.6%]), whose ages ranged from 20 to 67 years at T1 (*M* = 38.0 years, *SD* = 9.2). In terms of education level in T1, less than 0.2% had primary education, 0.3% had completed primary education, 1.7% had vocational education, 17.4% completed secondary education, 76.1% held a bachelor’s or master’s degree, and 4.3% had attained a doctoral degree. In terms of marital status, 52.2% were single, 37.8% were married, 9.4% were divorced, and 0.5% were widowed in T1.

## Procedure

An online survey study was conducted at two time points in Hungary. The first wave (T1) took place in spring 2018, while the second wave (T2) occurred exactly four years later in spring 2022. To collect data at T1, the survey was distributed through two prominent national news portals. To be eligible for participation, individuals had to be above 18 years old and currently employed. Before proceeding with the questionnaire on the Qualtrics platform, participants were presented with information regarding the study’s objectives and an estimated completion time. They provided their consent to participate, with the assurance of confidentiality and anonymity. The online survey included a variety of questions and assessments, encompassing items beyond the scope of this particular study. In total, it necessitated approximately 35 min to finish. Participation was entirely voluntary, and participants had the freedom to discontinue the survey at any point.

Upon completion of the T1 online questionnaire, participants were invited to express their willingness to take part in future research endeavors. If they agreed, they were requested to provide their email addresses. In spring 2022, during T2, the individuals who had previously provided their email addresses were contacted. Each participant received a unique code for accessing the survey. This follow-up questionnaire was shorter in length, taking approximately 10 min to complete. The rationale behind this decision was that during time T1, we assessed various psychological factors to determine their predictive role in work addiction at a later time. However, during time T2, we focused only on measuring work addiction.

The entire study, encompassing both waves of data collection, received approval from the institutional review board (IRB) of the research team’s university. Furthermore, the study adhered to the guidelines outlined in the Declaration of Helsinki.

## Measures

### Socio-demographic and work-related variables

The initial pages of both T1 and T2 online questionnaires included the following socio-demographic questions: age, sex, level of education, and marital status.

### Work addiction

For evaluating the symptoms of work addiction at T1 and T2, we employed the widely recognized Bergen Work Addiction Scale (BWAS) [66, 68]. This self-report scale was designed based on the ‘components model’ of addiction [69]. It consists of seven items, each assessing one of the seven core components of work addiction: salience, tolerance, mood modification, relapse, withdrawal, conflict, and problems. We used the Hungarian version of the scale, which has the same structure and demonstrates appropriate internal consistency [68]. Participants responded to each question using a 5-point scale, ranging from 1 (“never”) to 5 (“always”). The respondents were prompted to indicate their frequency of occurrence over the past year by answering the question, ‘How often during the last year have you.?’ An example item from the scale is: “Worked so much that it has negatively influenced your health?” The scale demonstrated high reliability in this sample (see Supplementary Material Table S1).

The developers of the scale [66] have established a cut-off point for work addiction, categorizing individuals as at-risk of work addiction if they respond “often” or “always” to at least four out of the seven items. To determine whether a participant exhibits a symptom, it is necessary to assess each item. Thus, if someone demonstrates at least four symptoms, they are considered having work addiction.

### Self-esteem

To measure global self-esteem at T1, we utilized the reliable and valid Hungarian version [70] of the Rosenberg Self-Esteem Scale (RSES), comprising 10 items [71]. The RSES is a self-reported scale where participants rate their agreement on a four-point Likert scale, ranging from “strongly agree” to “strongly disagree” (with scores ranging from 10 to 40). In this unidimensional scale, five items [2, 5, 6, 8, 9] were reversed, and they exhibited good internal consistency in the present sample (Table S1). An example item from the scale is: “At times I think I am no good at all.”

### Perfectionism

During T1, we employed the abbreviated version of the Multidimensional Perfectionism Scale (MPS) that assesses three dimensions of perfectionism [72, 73]. The Hungarian version was used, which demonstrates appropriate internal consistency [74]. This scale consists of 15 items, measuring Self-Oriented Perfectionism (SOP, five items; e.g., “I am perfectionistic in setting my goals.“), Other-Oriented Perfectionism (OOP, five items; e.g., “It doesn’t matter when someone close to me does not do their absolute best.“), and Socially Prescribed Perfectionism (SPP, five items; e.g., “My family expects me to be perfect.“). All the OOP subscale items are reversed. Respondents rated themselves on a seven-point Likert scale, ranging from “strongly agree” to “strongly disagree”. Each scale ranged from 5 to 35, and in our current sample, they all exhibited favorable reliability (Table S1).

### Narcissism

To assess narcissistic traits at T1, we utilized the Narcissistic Admiration and Rivalry Questionnaire Short Scale (NARQ-S) [75], which consists of six items. Since there is no validated Hungarian version of this scale, we conducted the translation ourselves. To adapt the questionnaire into Hungarian, bilingual experts translated it from English. Both translators were well-versed in the relevant terminology, with one being a native English speaker. Any differences in interpretation were resolved through discussion. Following this, an independent translator, a native English speaker with no prior exposure to the original version, performed a back-translation into English. Lastly, any remaining discrepancies were reviewed and corrected.

Participants were asked to rate themselves on a six-point Likert scale, ranging from 1 (“not agree at all”) to 6 (“agree completely”). The NARQ-S comprises two dimensions, Admiration (e.g., “I deserve to be seen as a great personality”) and Rivalry (e.g., “Most people are somehow losers”), each with three items. The Admiration subscale demonstrated good reliability within this sample. However, the internal consistency of the Rivalry subscale was poor (see Table S1).

### Psychopathological distress

We utilized two subscales, Anxiety and Depression, derived from the abbreviated 18-item version of the Brief Symptom Inventory (BSI-18) [76], to evaluate psychopathological distress at T1. The original BSI-18 comprises three subscales, each consisting of six items, targeting symptoms associated with depression, anxiety, and somatization. We used the Hungarian version, which follows the same structure and exhibits appropriate internal consistency [77]. To quantify psychopathological distress, in this study we computed a composite score by combining the depression and anxiety scales. Participants assessed the extent to which they experienced the symptom items during the previous week, rating each item on a 5-point scale that ranged from 1 (“not at all”) to 5 (“extremely”). An example item from the scale is: “Feeling so restless you couldn’t sit still”. The scale demonstrated excellent internal consistency within our sample (Table S1).

### Rumination

To evaluate rumination at T1, we employed the short version of the Ruminative Response Scale (RRS) [78]. This scale consists of 10 items, and respondents are asked to indicate what they feel sad, blue, or depressed about, what they typically do, or what they believe they should do. RRS assesses two types of rumination: Brooding (comprising five items, e.g., “Think' What am I doing to deserve this?‘”) and Reflective Pondering (comprising five items, e.g., “Write down what you are thinking and analyze it”). Brooding, viewed as a maladaptive facet of rumination, involves an unconstructive evaluation of an individual’s current unpleasant conditions. Conversely, Reflective Pondering is a more adaptive process of rumination characterized by deliberate engagement in cognitive problem-solving [78]. In our study, we used the Hungarian version, which has been found to be reliable and valid [79]. Respondents were asked to rate themselves on a four-point Likert scale, ranging from “almost never” to “almost always,” in response to the question “How often do you.“. Both subscales ranged from 5 to 20 and exhibited good reliability in the current sample (see Table S1).

### Work motivation

To evaluate various work motivations at T1, we utilized the Multidimensional Work Motivation Scale (MWMS) [80], which is grounded in the Self-Determination Theory proposed by Deci & Ryan [81]. The scale comprises 19 items, and respondents provide their answers using a 7-point response scale ranging from “not at all” to “completely” in response to the question “Why do you or would you put efforts into your current job?”. It encompasses six subscales that represent different work motivations: Extrinsic Regulation—Social (3 items; e.g., “To get others’ approval (e.g., supervisor, colleagues, family, clients…”), Extrinsic Regulation—Material (3 items; e.g., “Because I risk losing my job if I don’t put enough effort in it.”), Introjected Regulation (4 items; e.g., “Because otherwise I will feel ashamed of myself.”), Identified Regulation (3 items; e.g., “Because putting efforts in this job has personal significance to me.”), Intrinsic Motivation (3 items; e.g., “Because I have fun doing my job.”), and Amotivation (3 items; e.g., “I don’t know why I’m doing this job, it’s pointless work.”). As with the other scales, we used the Hungarian version, which demonstrates adequate reliability [82]. All the subscales showed good reliability in the current sample (see Table S1).

### Statistical analysis

All of the participants who took part in T2 answered all the questions, so there was no missing data in the final sample. However, those who did not participate in T2 were excluded from the analysis using listwise deletion; that is, respondents who only answered at T1 were removed from the analysis. Subsequently, we conducted a chi-square test and an independent sample t-test to compare individuals who dropped out at time T2 with those who remained in the study until time T2. We also conducted a preliminary analysis regarding gender, as we will perform separate latent class analyses for males and females. Additionally, we examined the gender invariance of the one-factor structure of the BWAS.

Following the basic statistical analysis (descriptives and bivariate correlations between the study variables), we categorized all individuals as either affected by work addiction or not, using the BWAS cut-off point. Subsequently, we grouped individuals into four categories based on their BWAS categorization at T1 and T2. These four groups were representative of the stability and variability of work addiction symptoms. To compare the four groups, we utilized ANOVA analysis for all the variables examined, and post-hoc LSD tests were employed to identify specific differences between groups. Additionally, we conducted multinomial regression analysis, with membership in the four groups as the outcome variable. In one model, personality variables served as predictors, while work motivation served as predictors in the other model, using the enter method of data entry.

We also conducted latent class analysis (LCA) to identify homogenous subgroups (latent classes) of participants based on their BWAS scores at both T1 and T2. Unlike the use of a cut-off score, this approach aims to identify groups of people who are similar to each other based on the empirical data (i.e. their BWAS scores at T1 and T2). Latent class analysis (LCA) was chosen as an alternative to group comparisons based on BWAS cut-off values. Using the designated cut-off values, it is possible to artificially delineate four distinct groups based on the two data points. In contrast, latent class analysis is a person-centered method that provides a statistically reliable approach for identifying groups of individuals based on their responses to certain variables. Individuals who exhibit similar responses (scores) are then classified into the same latent class. Thus, LCA is ideal for identifying latent subgroups within a population based on response patterns over time. In our study, it helped uncover distinct groups showing different work addiction trajectories, such as stability or change, across two time points, regardless of the cutoff point. Additionally, LCA treats class membership as a latent variable, offering a clearer view of the population’s diversity in work addiction patterns. This method also simplifies interpretation by grouping individuals into distinct classes, making it easier to understand and apply the findings.

Initially, we tallied the number of items participants responded to with “often” or “always” at both T1 and T2. This led to the derivation of ‘risk scores’ ranged from 0 to 7. These ‘risk scores’ from both T1 and T2 were utilized in LCA. The optimal number of classes was determined by examining models with different latent class counts, utilizing criteria such as the Akaike Information Criterion (AIC), Bayesian Information Criterion (BIC), Sample Size Adjusted Bayesian Information Criterion (SSA-BIC), and the Lo-Mendel-Rubin Adjusted Likelihood Ratio Test (LMRT). Smaller values of AIC, BIC, and SSA-BIC indicate better fit, and a non-significant LMRT suggests that adding an extra latent class would not improve the model fit. To enhance classification accuracy, we utilized the Entropy index, which achieves higher values (closer to 1) to indicate a more precise classification of participants (e.g., values around 0.8 represent high entropy) [83]. This analysis was performed separately for both genders, after we conducted independent samples t-tests to compare males and females across all variables. Subsequently, we compared the resulting classes based on covariates such as age, self-esteem, perfectionism, rumination, psychopathological distress, and motivations, utilizing the BCH method [84]. We then employed multinomial logistic regression analysis in a 3-step approach to investigate the relationship between the most likely latent class membership and covariates. This method was chosen because it allows for examining how various covariates predict membership in the latent classes identified through Latent Class Analysis (LCA). Multinomial logistic regression is ideal for modeling multiple categories (i.e., latent classes) simultaneously, providing insights into the factors differentiating class membership while controlling for the other covariates in the model. Data were analyzed using IBM SPSS statistics (Version 29) and Mplus 8.0 statistical software [85]. This study’s design and its analysis were not preregistered. All data and code for the analyses in our study are available at:

https://osf.io/7d468/?view_only=b0a9e3b0831e4fe09031e074c188a873.

## Results

### Preliminary analysis

The descriptive statistics of the variables for T1 and T2 are presented in Supplementary Materials Table S1, including ranges, means, and standard deviations.

Before proceeding with our main analyses, we examined whether there were any disparities in the socio-demographic attributes and variables between the drop-out participants and those who also participated in the T2 survey. Participants in both data collections are significantly older than dropouts. Among those who remained in the study, there are fewer individuals with secondary education and more with bachelor’s or master’s degree. Concerning the tested variables, the stayers exhibited significantly higher self-esteem, lower SOP and SPP. However, we found no differences between the two groups regarding all other variables (see Supplementary Materials Table S2).

We examined the gender invariance of the one-factor structure of the BWAS at T1 using a multigroup approach in Mplus 8.10. The results supported configural, metric, and scalar invariance across gender groups, with detailed findings presented in the Supplementary Material (Table S3).

### Group comparisons based on the BWAS cut-off scores

Based on their BWAS scores at T1 and T2 data collection, we categorized the participants into four groups: 1) the ‘Chronic WA group’ (CWA; N = 103; 17.58%)—those who were at-risk for WA both at T1 and T2; 2) the ‘Increased WA group’ (IWA; N = 37; 6.31%)—individuals who showed non-risk at T1 but at-risk at T2; 3) the ‘Recovered WA group’ (RWA; N = 124; 21.16%)—those who were at-risk at T1 but non-risk at T2; and 4) the ‘Permanently Non-WA group’ (PNW; *N* = 322; 54.95%)—those who were non-risk both at T1 and T2. First, we compared these four groups in descriptive variables at T1 and T2 (Supplementary Materials, Table S5). The four groups were not significantly different from each other in terms of gender, age, level of education or marital status.

The ANOVA analysis indicates that only on the NARQ-S admiration scale, no difference was found between the four groups (see Table 1). On the other-oriented perfectionism scale, only the RWA group and the PNW group showed a significant difference, with the permanently non-WA group scoring lower on OOP. Moreover, it was found that the CWA displayed the least favorable scores across all measures. Apart from the RWA, which had the lowest self-esteem at time T1, they also exhibited the highest SOP and reflective pondering scores. Additionally, the CWA group had significantly higher SPP, brooding, and psychopathological distress scores at T1 compared to all other groups (Table 1).

**Table 1:** Differences in personality and work motivations among the four groups based on BWAS categorization at T1 and T2

Variables at T1	(1) Chronic WA group (*N* = 103)	(2) Increased WA group (*N* = 37)	(3) Recovered WA group (*N* = 124)	(4) Permanently non-WA group (*N* = 322)	Difference *F* (df)
Self-esteem	26.53 (4.99)^2,4^	30.03 (5.31)^1,3^	27.63 (5.69)^2,4^	29.85 (5.22)^1,2^	13.14 (3)**
Self-Oriented Perfectionism	28.80 (5.49)^2,4^	25.46 (6.22)^3,4^	28.41 (5.06)^1,2^	24.60 (6.28)^1,3^	20.49 (3)**
Other-Oriented Perfectionism	20.89 (6.08)	19.57 (6.52)	21.66 (6.20)^4^	19.84 (5.83)^3^	3.18 (3)*
Socially Prescribed Perfectionism	20.99 (7.10)^2,3,4^	17.49 (7.43)^3,4^	17.60 (6.73)^1,4^	14.75 (5.98)^1,2,3^	25.96 (3)**
Narcissism– Admiration	10.39 (3.69)	11.41 (3.25)	10.75 (3.63)	10.33 (3.69)	1.37 (3)
Narcissism– Rivalry	9.25 (3.13)^4^	9.59 (3.65)^4^	9.14 (3.42)^4^	8.35 (3.01)^1,2,3^	4.07 (3)*
Rumination– Brooding	12.19 (3.13)^2,3,4^	10.08 (3.37)^1^	10.85 (3.10)^1,4^	9.71 (2.99)^1,3^	18.35 (3)**
Rumination– Reflection	11.91 (3.07)^4^	11.03 (3.48)	11.37 (3.21)^4^	10.21 (3.19)^1,3^	9.24 (3)**
Psychopathological Distress	28.69 (6.77) ^2,3,4^	22.97 (6.93)^1,3^	25.38 (6.46)^1,2,4^	21.81 (6.41)^1,3^	31.72 (3)**
Amotivation	6.55 (4.52)	6.46 (5.04)	6.08 (3.87)	7.02 (4.81)	1.35 (3)
Extrinsic social regulation	12.26 (5.43)^2,4^	10.41 (4.30)^1^	11.85 (4.98)^4^	9.67 (4.40)^1,3^	11.33 (3)**
Extrinsic material regulation	12.58 (5.34)^2,4^	10.40 (4.80)^1^	11.77 (5.53)^4^	10.22 (4.74)^1,3^	7.13 (3)**
Introjected regulation	21.15 (5.73)^4^	18.97 (5.55)	20.77 (5.31)^4^	17.24 (6.13)^1,3^	17.75 (3)**
Identified regulation	18.17 (3.27)^2,4^	16.62 (4.12)^1^	17.59 (2.89)^4^	15.69 (4.36)^1,3^	14.10 (3)**
Intrinsic motivation	14.34 (5.28)	14.70 (5.84)	15.06 (4.98)	13.77 (5.21)	1.97 (3)

Note. Superscript numbers indicate the groups that exhibit significant differences from one another. * *p* <.05; **p <.01

Regarding work motivation at T1, no significant differences between the four groups were found in either Amotivation or Intrinsic motivation. However, on the Extrinsic Social regulation scale, the CWA group scored significantly higher than all the other groups. Furthermore, on the extrinsic material, the introjected, and the identified regulation scales, both the CWA and the RWA groups scored the highest at T1 (Table 1).

### Prediction of belonging to work addiction groups by personality and motivations: multinomial regression analyses

Multinomial regression analysis using personality variables as predictors showed that four variables were significant predictors of group membership (see Table 2). For higher Self-Oriented Perfectionism at T1, compared to CWA group, there is a nearly 10% lower probability of being in the IWA group and a nearly 9% lower probability of being in the PNW group. In relation to SPP at T1, there is a 6% lower probability of being in the RWA group and a 7% lower probability of being in the PNW group, as compared to the CWA group. However, the rivalry score is 18% more likely to predict individuals who developed work addiction during the four years (IWA group) than those who showed the symptoms of work addiction both before and after the period (CWA group). Finally, individuals with higher levels of psychopathological distress at T1 are significantly 6% less likely to belong to the recovering group (RWA) and 7% less likely to belong to the non-WA (PNW) group, in comparison to chronic WA (CWA) (Table 2).

**Table 2:** Results of the multinomial logistic regression analysis involving personality variables predicting group membership

Variable	Comparison group	OR	*p*	95% CI
Self-esteem	Increased WA	1.027	0.648	0.916–1.151
	Recovered WA	0.928	0.056	0.860–1.002
	Permanently non-WA	0.973	0.456	0.906–1.045
**Self-Oriented Perfectionism**	**Increased WA**	**0.898**	**0.008**	**0.830–0.972**
	Recovered WA	1.006	0.845	0.946–1.070
	**Permanently non-WA**	**0.913**	**0.001**	**0.864–0.964**
Other-Oriented Perfectionism	Increased WA	0.952	0.176	0.887–1.022
	Recovered WA	1.006	0.793	0.959–1.056
	Permanently non-WA	0.979	0.356	0.937–1.024
**Socially Prescribed Perfectionism**	Increased WA	0.999	0.983	0.932–1.071
	**Recovered WA**	**0.941**	**0.011**	**0.897–0.986**
	**Permanently non-WA**	**0.931**	**0.002**	**0.891–0.973**
Narcissism– Admiration	Increased WA	1.041	0.596	0.896–1.210
	Recovered WA	1.024	0.650	0.924–1.134
	Permanently non-WA	0.986	0.764	0.896–1.084
**Narcissism– Rivalry**	**Increased WA**	**1.185**	**0.026**	**1.020–1.377**
	Recovered WA	1.031	0.565	0.929–1.144
	Permanently non-WA	1.074	0.151	0.974–1.183
Rumination– Brooding	Increased WA	0.933	0.438	0.782–1.112
	Recovered WA	0.939	0.289	0.835–1.055
	Permanently non-WA	0.978	0.696	0.877–1.091
Rumination - Reflective Pondering	Increased WA	1.018	0.790	0.891–1.164
	Recovered WA	1.010	0.832	0.918–1.112
	Permanently non-WA	0.959	0.359	0.878–1.049
**Psychopathological Distress**	**Increased WA**	**0.900**	**0.028**	**0.819–0.989**
	Recovered WA	0.940	0.051	0.884-1.000
	**Permanently non-WA**	**0.882**	**< 0.001**	**0.832–0.935**

Note. Reference category: Chronic work addiction group. Statistically significant effects are shown in bold (*p* <.05)

Among work motivations, only extrinsic material and identified regulations at T1 emerged as significant predictors of group membership. In comparison to the chronic WA group, higher levels of extrinsic material regulation at time T1 decrease the odds of belonging to the PNW group by almost 7%. However, identified regulation was found to be a stronger predictor. A higher level of this motivation at T1 reduces the odds of being in the PNW group by almost 20%, compared to CWA individuals. It also reduces the odds of being in the recovering group (RWA) by 12% (see Table 3).

**Table 3:** Results of the multinomial logistic regression analysis involving motivations predicting group membership

Motivation	Comparison group	OR	*p*	95% CI
Amotivation	Increased WA	0.967	0.566	0.861–1.085
	Recovered WA	0.966	0.402	0.891–1.047
	Permanently non-WA	0.964	0.313	0.898–1.035
Extrinsic social regulation	Increased WA	0.976	0.641	0.883–1.080
	Recovered WA	1.007	0.843	0.940–1.079
	Permanently non-WA	0.955	0.138	0.898–1.015
**Extrinsic material regulation**	Increased WA	0.850	0.930	0.850–1.016
	Recovered WA	0.967	0.280	0.910–1.028
	**Permanently non-WA**	**0.932**	**0.011**	**0.883–0.984**
Introjected regulation	Increased WA	0.908	0.993	0.908–1.087
	Recovered WA	1.014	0.675	0.950–1.083
	Permanently non-WA	0.964	0.200	0.911–1.020
**Identified regulation**	Increased WA	0.957	0.705	0.705–0.957
	**Recovered WA**	**0.882**	**0.033**	**0.785–0.990**
	**Permanently non-WA**	**0.807**	**< 0.001**	**0.728–0.894**
Intrinsic motivation	Increased WA	1.166	0.944	0.944–1.166
	Recovered WA	1.038	0.308	0.966–1.114
	Permanently non-WA	1.031	0.337	0.968–1.099

Note. Reference category: Chronic work addiction group. Statistically significant effects are shown in bold (*p* <.05)

### Latent class analysis

**Fig. 1:**
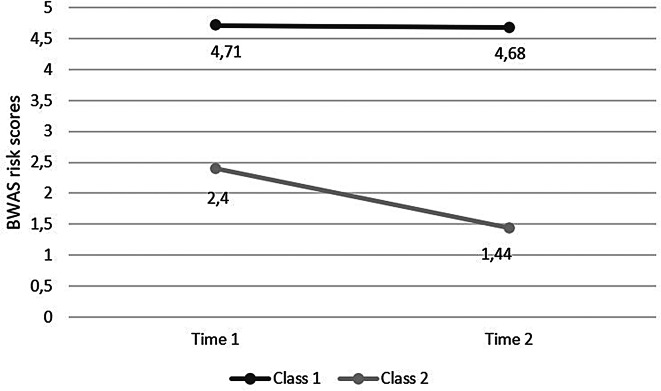
Two latent classes of work addiction risk scores in the total sample (*N* = 586). Note. BWAS, bergen work addiction scale

The latent class analysis was conducted based on the risk categorization performed on the BWAS items at both T1 and T2. The LMRT of the 3-class model was no longer significant (*p* =.114), suggesting the two-cluster over the three-cluster solution. Additionally, the 2-class model has higher entropy value (0.821), which suggests an accurate classification of the individuals (see Table 4). Moreover, the LMRT of the 3-class model ceased to be statistically significant (*p* =.114), indicating a preference for the two-class model over the three-class model (see Table 4). Class 1 (*N* = 160; 27.3%) exhibited elevated risk scores at both waves (*M*_T1_ = 4.71, *SD*_T1_ = 1.61; *M*_T2_ = 4.68, *SD*_T2_ = 1.07), whereas Class 2 (*N* = 426; 73.7%) displayed lower risk scores across both waves (*M*_T1_ = 2.40, *SD*_T1_ = 1.61; *M*_T2_ = 1.44, *SD*_T2_ = 1.07) (see Fig. 1). Given the LCA outcomes, we can distinguish between a persistently WA group and a persistently non-WA group. This observation implies that the LCA conducted on the complete dataset did not yield groups exhibiting shifts in work addiction symptoms over time. Consequently, we refrained from contrasting these groups in terms of personality traits and motivations, opting to proceed with gender-based comparisons instead.

**Table 4:** Fit indices for the latent class analysis of risk scores (*N* = 586)

	AIC	BIC	SSA-BIC	Entropy	LMRT	*p*
**2-class model**	**4510.4**	**4541.0**	**4518.8**	**0.821**	**251.8**	**< 0.001**
3-class model	4458.4	4502.2	4470.4	0.743	55.1	0.114

Note. AIC, Akaike Information Criteria; BIC, Bayesian Information Criteria; SSA-BIC, Sample Size Adjusted Bayesian Information Criteria; LRT, Lo-Mendell-Rubin Adjusted Likelihood Ratio Test. The selected model is shown in bold

### Latent class analysis by gender

Drawing from gender disparities in work addiction evident in representative population studies, we replicated the latent class analysis for each gender. Additionally, our own univariate analyses using independent samples t-tests also revealed significant gender differences (see Supplementary Table S4). As shown, women scored significantly higher than men on the work addiction scale measured at both T1 and T2, as well as on the self-oriented and socially prescribed perfectionism scales, the anxiety symptoms scale, the brooding and reflective pondering rumination scales, and the introjected and identified regulation scales among the motivation measures.

**Fig. 2:**
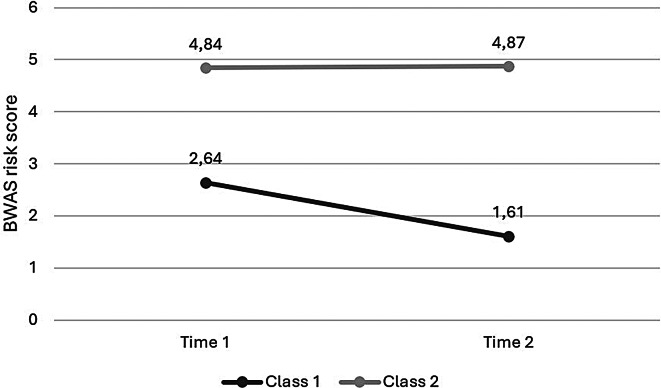
Two latent classes of work addiction risk scores in females (*N* = 301). *Note*. BWAS, bergen work addiction scale

Conducting the latent class analysis, we found that, just as in the entire sample, the two-class model exhibited the best fit among females (Table 5). The LMRT for the three-class model was no longer significant (*p* =.080), suggesting that the two-class solution was preferable over the three-class solution. Additionally, based on the higher entropy value (0.829), the two-class model classified individuals more accurately (see Table 5). We discovered that Class 1 (*N* = 222; 73.8%) showed low risk scores in both time points (*M*_T1_ = 2.64, *SD*_T1_ = 1.61; *M*_T2_ = 1.61, *SD*_T2_ = 1.07), while Class 2 (*N* = 79; 26.2%) displayed high risk scores at both time points (*M*_T1_ = 4.84, *SD*_T1_ = 1.61; *M*_T2_ = 4.87, *SD*_T2_ = 1.07) (Fig. 2). That is, the LCA produced a chronic WA group (CWA) and a permanently non-WA group (PNW) among females.

**Table 5:** Fit indices for the latent class analysis of work addiction risk scores in females (*N* = 301)

	AIC	BIC	SSA-BIC	Entropy	LMRT	*p*
**2-class model**	**2329.2**	**2355.1**	**2332.9**	**0.829**	**120.8**	**< 0.001**
3-class model	2313.5	2350.6	2318.9	0.738	20.5	0.080

Note. AIC, Akaike Information Criteria; BIC, Bayesian Information Criteria; SSA-BIC, Sample Size Adjusted Bayesian Information Criteria; LRT, Lo-Mendell-Rubin Adjusted Likelihood Ratio Test. The selected model is shown in bold

**Fig. 3:**
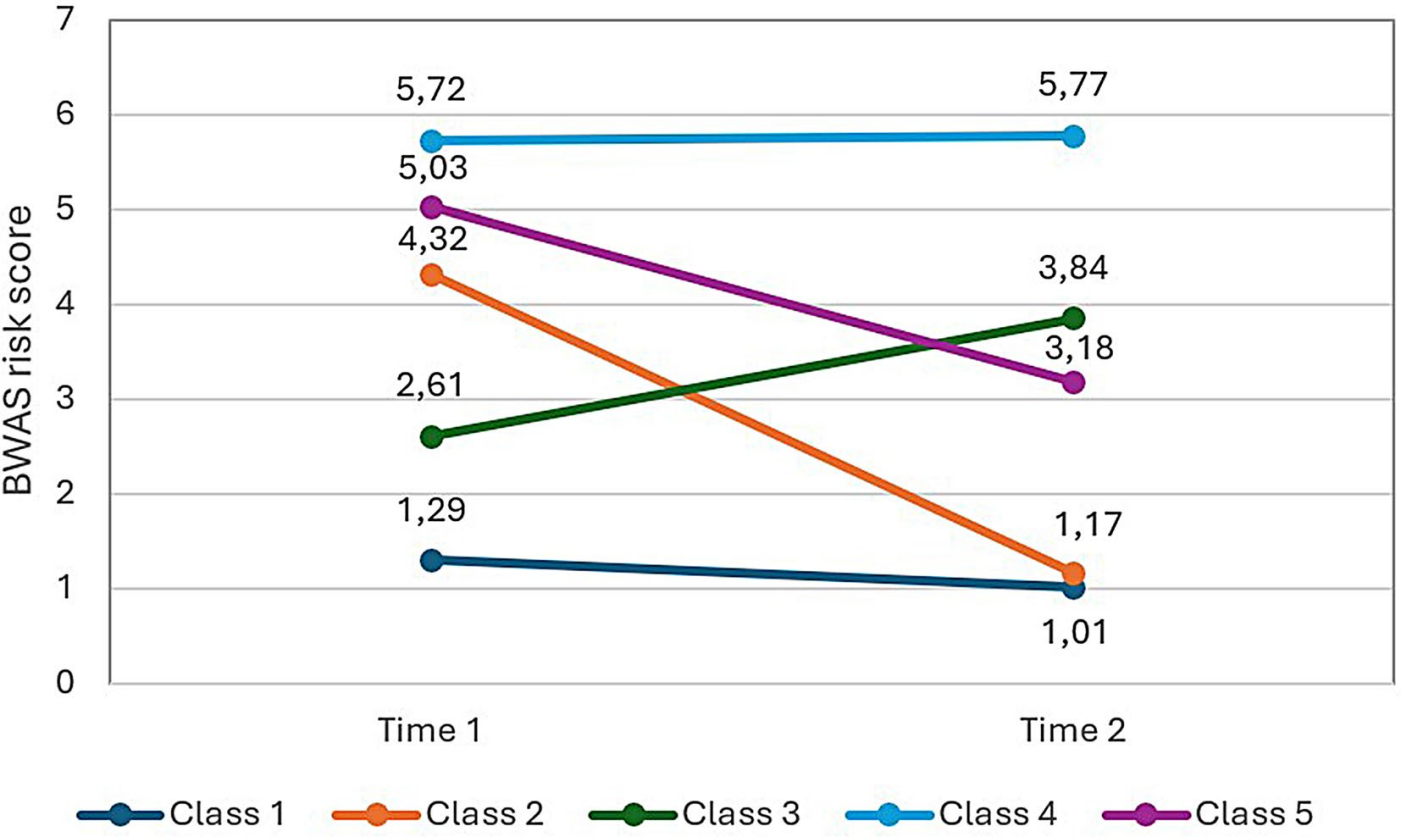
Five latent classes of work addiction risk scores in males (*N* = 285). *Note*. BWAS, Bergen Work Addiction Scale

In contrast to females, the five-class model showed the most appropriate fit in males (Table 6). Based on the non-significant LMRT value (*p* =.143) for the six-class model, the five-class model was chosen. This model showed high entropy (> 0.8), also suggesting an accurate classification of individuals. Class 1 (*N* = 152, 53.3%; *M*_T1_ = 1.29, *SD*_T1_ = 0.87; *M*_T2_ = 1.01, *SD*_T2_ = 0.80) displayed consistently low scores on BWAS, characterizing the ‘permanently non-WA’ group (PNW). Respondents in Class 2 (*N* = 36, 12.6%; *M*_T1_ = 4.32, *SD*_T1_ = 0.87; *M*_T2_ = 1.17, *SD*_T2_ = 0.80) exhibited a substantial decrease in BWAS scores, leading to the designation of the ‘strong recovered WA’ group (SRWA). Similarly, members of Class 5 (*N* = 38, 13.3%; *M*_T1_ = 5.03, *SD*_T1_ = 0.87; *M*_T2_ = 3.18, *SD*_T2_ = 0.80) displayed a decline in the BWAS symptoms, although to a lesser extent. They correspond to the ‘light recovered WA’ group (LRWA). Class 3 (*N* = 35, 12.2%; *M*_T1_ = 2.61, *SD*_T1_ = 0.87; *M*_T2_ = 3.18, *SD*_T2_ = 0.80), titled ‘increased WA’ group (IWA), portrayed a progression of work addiction symptoms over time, with a slight rise in the risk score. Class 4 (*N* = 24, 8.4%; *M*_T1_ = 5.72, *SD*_T1_ = 0.87; *M*_T2_ = 5.77, *SD*_T2_ = 0.80), representing the ‘chronic WA’ (CWA) group, consistently demonstrated elevated work addiction scores both at T1 and T2 (Fig. 3).

**Table 6:** Fit indices for the latent class analysis of work addiction risk scores in males (*N* = 285)

	AIC	BIC	SSA-BIC	Entropy	LMRT	*p*
2-class model	2176.4	2201.9	2179.7	0.819	133.7	< 0.001
3-class model	2142.3	2178.8	2147.1	0.846	37.8	0.029
4-class model	2099.0	2146.5	2105.3	0.848	46.5	< 0.001
**5-class model**	**2086.1**	**2144.6**	**2093.8**	**0.844**	**17.8**	**0.035**
6-class model	2079.7	2149.1	2088.9	0.831	11.7	0.143

Note. AIC, Akaike Information Criteria; BIC, Bayesian Information Criteria; SSA-BIC, Sample Size Adjusted Bayesian Information Criteria; LRT, Lo-Mendell-Rubin Adjusted Likelihood Ratio Test. The selected model is shown in bold

### Differences in personality and motivation among gender-differentiated work addiction groups

As a first step, a comparison was conducted between the two classes of females across the tested variables by the means of BCH method implemented in Mplus. This analysis revealed that the CWA group exhibited significantly lower self-esteem, yet significantly higher scores in SOP, SPP, brooding, reflective pondering, and psychological distress, when compared to the PNW group. Concerning work motivations, the CWA group of females displayed significantly elevated scores in extrinsic material, introjected, and identified regulations, whereas the remaining regulations were not distinct from those of PNW group (Table 7).

**Table 7:** Group comparison of work addiction risk score groups in females

	Class 1 Permanently Non-WA group Mean (SE)	Class2 Chronic WA group Mean (SE)	Wald	*p*
Age	38.30 (0.66)	37.87 (1.13)	0.1	0.752
**Self-Esteem**	**29.18 (0.38)**	**25.52 (0.63)**	**11.9**	**< 0.001**
**Self-Oriented Perfectionism**	**26.01 (0.42)**	**28.71 (0.74)**	**9.3**	**0.002**
Other-Oriented Perfectionism	20.66 (0.43)	19.96 (0.82)	0.5	0.465
**Socially Prescribed Perfectionism**	**16.00 (0.47)**	**21.44 (0.90)**	**26.4**	**0.001**
Narcissism– Admiration	10.72 (0.24)	10.50 (0.45)	0.2	0.692
Narcissism - Rivalry	8.51 (0.21)	8.79 (0.34)	0.4	0.505
**Rumination– Brooding**	**10.33 (0.22)**	**12.26 (0.36)**	**19.2**	**< 0.001**
**Rumination - Reflective Pondering**	**11.06 (0.23)**	**12.35 (0.37)**	**8.2**	**0.004**
**Psychopathological Distress**	**23.32 (0.47)**	**27.84 (0.82)**	**20.8**	**< 0.001**
Amotivation	6.65 (0.32)	6.58 (0.61)	0.0	0.918
Extrinsic Social	10.59 (0.34)	11.99 (0.68)	3.1	0.077
**Extrinsic Material**	**10.41 (0.36)**	**12.09 (0.68)**	**4.3**	**0.037**
**Introjected Regulation**	**18.72 (0.40)**	**21.97 (0.55)**	**20.7**	**< 0.001**
**Identified Regulation**	**16.66 (0.29)**	**18.40 (0.39)**	**11.628**	**< 0.001**
Intrinsic	13.99 (0.38)	14.21 (0.65)	0.1	0.791

Note. SE: standard error. Statistically significant differences are shown in bold

In the multinomial regression analysis, with age as a controlling factor, only the variables that produced significant outcomes in the previous (BHC) analysis were incorporated. Among these, SPP and identified regulation emerged as substantial predictors in the regression analysis. This implies an elevated T1 SPP score increases the probability of belonging to the CWA group in females by nearly 8%. Similarly, a higher previously assessed identified regulation score elevates the odds of being in the CWA group by nearly 20% among females (Table 8).

**Table 8:** Multinomial logistic regression analysis for predicting chronic work addiction group in females (*N* = 299*)

Covariates	Permanently non-WA group OR [95% CI]	*p*
Age	0.99 [0.95–1.03]	0.567
Self-Esteem	1.03 [0.94–1.12]	0.556
Self-Oriented Perfectionism	1.01 [0.92 − 0.11]	0.797
**Socially Prescribed Perfectionism**	**0.93 [0.87–0.98]**	**0.007**
Psychopathological Distress	0.96 [0.89–1.03]	0.249
Rumination– Brooding	0.95 [0.83–1.09]	0.453
Rumination– Reflective Pondering	0.95 [0.85–1.07]	0.436
Extrinsic Material Regulation	0.99 [0.92–1.07]	0.866
Introjected Regulation	0.97 [0.88–1.07]	0.567
**Identified Regulation**	**0.84 [0.71–0.99]**	**0.037**

Note. Reference category: Chronic WA group *: two participants did not respond to one of the covariates. Statistically significant effects are shown in bold

BCH analysis was also conducted for the five classes identified among males, comparing the groups in terms of personality and work motivations. We found significant differences in SOP, SPP, rivalry, reflective pondering, brooding, psychopathological distress, and four motivation subscales (extrinsic social, extrinsic material, introjected regulation, and identified regulation) (see Table 9). Post-hoc analysis unveiled consistent distinctions between the PNWA group and both the CWA and LRWA groups across these variables. The CWA and LRWA groups exhibited lower self-esteem, higher perfectionism, increased rumination, heightened psychopathological distress, and motivation scores. Notably, no differences were observed between the CWA and LRWA groups across any of the measurements. Additionally, the PNWA and IWA groups did not exhibit any significant differences in the measurements. However, in comparison to the CWA and LRWA groups, the IWA group demonstrated elevated self-esteem, reduced perfectionism, diminished psychopathological distress, and lower scores in extrinsic material and introjected regulation. Further post-hoc analysis indicated that the SRWA group, in contrast to the CWA group, presented a favorable profile: participants in this groups reported lower self-oriented and socially prescribed perfectionism, decreased brooding, diminished psychopathological distress, and higher identified regulation.

**Table 9:** Group comparison of work addiction risk score groups in males (*N* = 284)

	Class 1 PNW group Mean (SE)	Class2 SRWA group Mean (SE)	Class 3 IWA group Mean (SE)	Class 4 CWA group Mean (SE)	Class 5 LRWA group Mean (SE)	Wald	*p*	Significant paired post hoc tests
**Age**	39.11 (0.79)	34.82 (1.41)	39.02 (1.76)	36.53 (1.53)	35.34 (2.08)	**10.5**	**0.032**	C1 vs. C2
**Self-Esteem**	31.02 (0.44)	27.68 (0.90)	30.43 (0.99)	25.97 (0.99)	24.70 (1.20)	**49.7**	**< 0.001**	C1 vs. C2, C4, C5 C2 vs. C3 C3 vs. C4, C5
**Self-Oriented Perfectionism**	23.76 (0.57)	26.23 (1.21)	25.29 (0.99)	30.93 (0.74)	29.10 (0.99)	**70.7**	**< 0.001**	C1 vs. C4, C5 C2 vs. C4 C3 vs. C4, C5
Other-Oriented Perfectionism	19.51 (0.50)	20.86 (1.18)	19.93 (1.07)	23.02 (0.41)	21.36 (1.14)	7.7	0.103	
**Socially Prescribed Perfectionism**	13.64 (0.50)	16.25 (1.16)	15.66 (1.06)	22.02 (1.43)	19.25 (1.25)	**47.6**	**< 0.001**	C1 vs. C2, C4, C5 C2 vs. C4 C3 vs. C4, C5
Narcissism– Admiration	10.35 (0.29)	10.42 (0.67)	11.13 (0.63)	11.01 (0.76)	8.78 (0.77)	5.7	0.222	
**Narcissism– Rivalry**	8.26 (0.28)	8.96 (0.61)	9.65 (0.73)	10.50 (0.76)	9.66 (0.77)	**12.3**	**0.015**	C1 vs. C4
**Psychopathological Distress**	20.72 (0.54)	23.62 (1.02)	21.21 (1.32)	31.21 (1.45)	28.66 (1.42)	**71.9**	**< 0.001**	C1 vs. C2, C4, C5 C2 vs. C4, C5 C3 vs. C4, C5
**Rumination– Brooding**	9.06 (0.25)	10.44 (0.56)	9.67 (0.68)	12.31 (0.68)	11.49 (0.70)	**32.2**	**< 0.001**	C1 vs. C2, C4, C5 C2 vs. C4 C3 vs. C4
**Rumination– Reflective Pondering**	9.63 (0.28)	11.12 (0.52)	9.22 (0.61)	11.94 (0.62)	11.04 (0.63)	**20.0**	**0.001**	C1 vs. C2, C4, C5 C2 vs. C3 C3 vs. C4
Amotivation	7.12 (0.42)	6.90 (0.86)	6.07 (0.88)	5.74 (0.65)	6.81 (0.77)	3.8	0.438	
**Extrinsic Social Regulation**	9.26 (0.38)	12.04 (0.91)	9.21 (0.77)	13.11 (1.11)	11.55 (0.90)	**21.5**	**< 0.001**	C1 vs. C2, C4, C5 C2 vs. C3 C3 vs. C4
**Extrinsic Material Regulation**	10.20 (0.41)	12.07 (0.10)	9.99 (0.87)	13.11 (0.99)	13.26 (1.16)	**15.6**	**0.004**	C1 vs. C4, C5 C3 vs. C4, C5
**Introjected Regulation**	16.20 (0.56)	19.53 (1.18)	16.48 (1.19)	22.12 (1.13)	21.62 (1.34)	**36.4**	**< 0.001**	C1 vs. C2, C4, C5 C3 vs. C4, C5
**Identified Regulation**	15.11 (0.38)	16.48 (0.64)	15.89 (0.67)	18.71 (0.64)	17.39 (0.67)	**28.2**	**< 0.001**	C1 vs. C4, C5 C2 vs. C4 C3 vs. C4
Intrinsic Motivation	13.92 (0.43)	14.04 (0.98)	14.48 (1.03)	15.24 (1.06)	15.75 (1.10)	3.7	0.446	

Note. SE: standard error. PNW, Permanently Non-Work Addiction group; SRWA, Strong Recovered Work Addiction group; IWA, Increased Work Addiction group; CWA, Chronic Work Addiction group; LRWA, Light Recovered Work Addiction group. Statistically significant differences are shown in bold

In the multinomial regression analysis, while controlling for age, we once again included only the variables that yielded significant results in the previous (BHC) analysis (Table 10). When compared to the chronic WA group, reduced psychopathological distress was linked with the membership in the PNWA, SRWA, and IWA groups. Furthermore, in comparison to the chronic WA group, diminished levels of self-oriented and socially prescribed perfectionism, as well as and lower identified regulation, were associated with belonging to the PNWA group. However, no statistically significant outcomes were observed when estimating LRWA membership.

**Table 10:** Multinomial logistic regression analysis for predicting chronic work addiction group in males (*N* = 284*)

	Class 1 Permanently non-WA group		Class 2 Strong recovered WA group		Class 3 Increased WA group		Class 5 Light recovered WA group	
	OR [95% CI ]	*p*	OR [95% CI ]	*p*	OR [95% CI ]	*p*	OR [95% CI ]	*p*
Age	1.01 [0.94–1.02]	0.804	0.96 [0.88–1.05]	0.412	1.00 [0.91–1.10]	0.956	0.97 [0.84–1.12]	0.686
Self-esteem	1.02 [0.81–1.29]	0.879	0.86 [0.68–1.08]	0.193	0.99 [0.77–1.28]	0.944	0.82 [0.63–1.06]	0.127
**Self-Oriented Perfectionism**	**0.83 [0.69–0.99]**	**0.046**	0.84 [0.70–1.01]	0.064	0.84 [0.67–1.06]	0.148	0.93 [0.75–1.16]	0.508
**Socially Prescribed Perfectionism**	**0.86 [0.76–0.98]**	**0.024**	0.87 [0.73–1.04]	0.112	0.93 [0.77–1.12]	0.432	0.86 [0.71–1.10]	0.273
Narcissism– Rivalry	1.06 [0.83–1.34]	0.644	1.05 [0.82–1.34]	0.695	1.25 [0.93–1.69]	0.146	0.97 [0.73–1.30]	0.856
**Psychopathological Distress**	**0.77 [0.62–0.95]**	**0.014**	**0.76 [0.61–0.94]**	**0.012**	**0.75 [0.60–0.93]**	**0.009**	0.86 [0.63–1.19]	0.363
Rumination– Brooding	1.09 [0.84–1.41]	0.540	1.06 [0.76–1.47]	0.733	1.22 [0.87–1.71]	0.248	1.00 [0.68–1.49]	0.984
Rumination– Reflective Pondering	1.02 [0.79–1.31]	0.900	1.08 [0.80–1.48]	0.610	0.92 [0.67–1.25]	0.576	0.97 [0.75–1.26]	0.820
Extrinsic Social Regulation	0.99 [0.82–1.21]	0.942	1.10 [0.89–1.35]	0.381	0.94 [0.76–1.16]	0.564	0.99 [0.79–1.24]	0.924
Extrinsic Material Regulation	1.01 [0.86–1.19]	0.876	1.04 [0.86–1.25]	0.713	0.99 [0.82–1.20]	0.928	1.07 [0.87–1.31]	0.539
Introjected Regulation	1.02 [0.89–1.16]	0.792	1.01 [0.84–1.21]	0.920	0.98 [0.81–1.17]	0.794	1.10 [0.75–1.61]	0.616
**Identified Regulation**	**0.74 [0.56–0.97]**	**0.026**	0.86 [0.61–1.24]	0.428	0.85 [0.64–1.14]	0.272	0.87 [0.61–1.23]	0.429

Note. Reference category: Chronic WA group *: one participant did not respond to one of the covariates

## Discussion

Despite increasing research on work addiction, little is known about why some individuals develop chronic work addiction while others do not. The present longitudinal study aimed to address this gap by examining how personality traits and work motivation predict changes in work addiction symptoms over a four-year period among adult workers, with a focus on gender differences.

Using both cut-off-based grouping and latent class analysis, we consistently identified a group with persistently high work addiction scores. Across genders, those chronically addicted to work exhibited lower baseline self-esteem, higher socially prescribed perfectionism, brooding, psychopathological distress, and stronger extrinsic and introjected work motives. Multinomial regression analyses showed that chronic work addiction was predicted by earlier self-oriented and socially prescribed perfectionism, psychopathological distress, lower narcissistic rivalry, and particularly by identified and extrinsic material regulation.

On the one hand, these findings suggest that work addiction can be a long-lasting, persistent problem and, contrary to previous misconceptions, does not necessarily disappear over time [86], at least for a certain subgroup of individuals, as identified through the latent class analysis, which revealed a chronically work-addicted group. Moreover, both some personality traits and specific work motivations increase the likelihood that a person will not recover from work addiction. This certainly provides evidence that work addiction is not only determined by environmental factors (e.g., organizational characteristics) [87, 88] but that personality as a risk factor plays a role in the persistence of the problem. Perfectionism, both self-oriented and especially socially prescribed, is a personality trait that should be highlighted. The high, even unrealistically high expectations that individuals have of themselves, or perceive others to have of them, keep them in a permanent state of work addiction, as they can never fully meet these expectations. This is likely to lead them to work more and more to prove themselves to themselves and others. Our results confirm the hypothesis of several previous theories of work addiction regarding the paramount importance of perfectionism [2, 29] and draw attention to the fact that perfectionism as a transdiagnostic risk factor [89] also affects work addiction. Thus, work addiction can be paralleled with a number of clinically diagnosed conditions, such as certain eating disorders, affective disorders, or personality disorders [90], where the role of socially prescribed perfectionism is also significant. This aligns with our finding that chronic work addiction is also predicted by psychological distress. Being stuck in work addiction is therefore an indication of psychological difficulties in individuals, rather than an indication that the enjoyment of or involvement in work keeps them compulsively [91]. Our findings on work motivation also support this notion. Intrinsic work motivation is not a predictor of work addiction, as all our analyses have clearly demonstrated, consistent with previous cross-sectional research [52]. In contrast, those unable to break free from work addiction do so for material reasons but are even more driven by identified regulation. In other words, for them, working is perceived as personally significant, important, and aligned with their individual values. Here, we can even observe the salience element of the component model of addiction [23, 69], where work stands out from other activities due to its personal importance to the individual.

When interpreting the results, it is important to consider the methodological issue that a significant number of participants did not complete the questionnaire at T2. These dropout individuals were characterized by lower self-esteem and higher levels of both self-oriented and socially prescribed perfectionism. The fact that the remaining sample consisted of individuals with higher self-esteem and lower perfectionism suggests that those carrying the greatest psychological burdens (i.e., low self-esteem and high perfectionism) were more likely to drop out. Consequently, the most vulnerable group may be underrepresented in the final analysis, potentially leading to an underestimation of the chronic nature of work addiction in the broader population. Additionally, it is possible that individuals most affected by work addiction were more likely to drop out due to their workload (e.g., they may have been too overburdened to participate in the second wave of data collection). If this is the case, the prevalence of chronic work addiction may also be underestimated in our final sample. Moreover, the fact that individuals with high levels of perfectionism were more likely to drop out raises the question of whether those who persist in chronic work addiction may employ different coping mechanisms or be less affected by certain perfectionist traits. Furthermore, given that dropout individuals had higher levels of perfectionism, it is possible that our identified predictors are somewhat distorted. If we had been able to account for the full initial sample, perfectionism might have emerged as an even stronger predictor of the chronicity of work addiction. Due to these limitations, the generalizability of our findings should be approached with caution.

Considering that several representative normal population surveys have revealed significant gender differences and that there are also gender disparities in the personality dimensions we are examining, we deemed it appropriate to conduct an exploratory latent class analysis, separately examining the two genders. It is important to note, however, that in this case, the sample sizes were smaller, so the results should be interpreted with caution. Latent class analyses revealed an important difference between the two genders. While men showed more variation in work addiction over time, with five different groups identified according to whether symptoms of work addiction decreased, increased, or remained stable, only two groups emerged for women. Among females, one group consisted of permanently non-WA individuals, and the other group was defined as chronic WA individuals. It therefore appears to be a more stable problem or disorder in women than in men. What is more, a higher proportion of women were found to be chronically work addicted (26.2% were in the CWA group) than men (only 8.4% were in the CWA group). However, this difference is also attributed to the sample being fragmented into five groups for men. Looking at the main findings of our research summarized above, it emerges that there are general gender differences in the traits identified as risk factors (such as self-esteem, perfectionism, and rumination). Women are more likely than men to ruminate [92] and several studies suggest that they are more likely to have a higher socially prescribed perfectionism [93], but also lower self-esteem [60]. Univariate analyses show that women also exhibit the same individual risk factors for work addiction as the total sample, except for narcissistic rivalry. Thus, rivalry does not contribute to the persistence of work addiction in women, which is not surprising given that women as a whole are less likely to possess this trait than men [94]. However, multivariate analysis revealed only a significant predictive role for socially prescribed perfectionism and identified regulation in women. On the other hand, in men, other personality traits also proved to be significant predictors. A higher level of self-oriented perfectionism and psychological distress also increases the chance that someone will remain permanently addicted to work. This is in addition to socially prescribed perfectionism and identified regulation, which were also significant predictors here, as they were in women. It appears that men are more inclined towards work addiction than women in their pursuit of meeting their own high expectations, not just those of others. Also, it is conceivable that they use work as a tool to “treat” their psychological symptoms more than women. However, for this, we would need to know more about the motive of work as escapism or coping mechanism in work addiction; studies related to this have not yet been published. In the case of other behavioral addictions, such as gambling disorder or gaming disorder, these motivations play key roles in the presence of symptoms [95, 96]. These motivations should definitely be investigated in work addiction in the future, and gender differences can be very relevant from this point of view. At the same time, the relationship between psychological distress and chronic work addiction may also indicate that more psychologically vulnerable individuals have reduced capacity to change their lives or recover from addiction. This pattern is consistent with other addictions, where associated psychological disorders tend to complicate the recovery process [97]. It appears that this is no different in the case of work addiction.

When considering gender differences, it’s important to note that distinct trajectories emerged for each gender: while only two groups appeared for women, five distinct groups were found for men. The potential reasons behind these differences should be examined. Traditional gender roles may influence how individuals experience work addiction and how susceptible they are to change. Men may be more socially motivated to view their work as a central factor in their success and self-esteem [98], which could explain why some of their groups exhibit significant changes (e.g., recovery or deterioration). In contrast, the presence of two stable groups among women (CWA and PNW) suggests that work addiction is either deeply ingrained (and remains chronic) or does not develop at all. Another possible explanation is that men’s workplace mobility, promotion opportunities, and career dynamics may differ from those of women [99, 100], which could influence the temporal patterns of their work addiction. For instance, if men are more likely to switch jobs or career phases, this could contribute to the varying addiction patterns. In contrast, for women, due to the need to balance work and family life, careers may be more stable or predictable, which may result in less fluctuation in work addiction over time. Related to this, workplace stress among women often stems not only from job performance but also from the imbalance between work and private life [101]. If someone is continuously overburdened, the likelihood of their work addiction pattern changing over time decreases. This could explain why women tend to show either persistently low or persistently high levels of work addiction, with fewer instances of improvement or deterioration.

It is also possible that work addiction in women might be less related to external factors (e.g., competition, career progression) and more strongly related to stable internal psychological traits. In this case, tendencies toward work addiction may change less over time, which could explain why women show less variability in their trajectories. Due to different role expectations, men’s work addiction may be linked to competition and performance orientation [102, 103], whereas in women, it may be more associated with self-imposed expectations and the pressure of caregiving roles [104, 105]. If men engage in work addiction for multiple reasons (e.g., performance pressure, financial security, status), this could account for the greater heterogeneity. In contrast, in women, work addiction may manifest as a persistent internal expectation, leading to less variability in its temporal patterns. Additionally, workplace structures and gender roles may also limit the extent to which work addiction patterns can change among women. Restricted access to leadership positions, the glass ceiling effect, and lower workplace autonomy [106, 107] may result in workplace stress becoming “entrenched” for female employees, contributing to more uniform and less differentiated work addiction groups. These explanations undoubtedly require further targeted research.

If we consider our findings from the perspective of protecting against work addiction or promoting recovery, we should emphasize similar individual dimensions as those related to potential risk factors. Our results indicate that individuals who initially reported higher levels of by work addiction but no longer exhibited symptoms after four years tended to report lower levels of socially prescribed perfectionism, less rumination on negative events, lower psychological distress, and lower levels of identified regulation compared to those who continued to show symptoms. However, our examination primarily focuses on the risk factors in personality and motivation that may contribute to work addiction. There are undoubtedly several other factors yet unknown that warrant investigation. When we consider other types of addiction, we observe that spontaneous recovery often involves various factors such as changes in life circumstances, social pressure, the development of health or mental health issues, or other negative events that serve as turning points in a person’s life [108–110]. These factors may also play a role in recovering from work addiction, an area that lacks sufficient research and investigation in our current study. Additionally, it is possible that some participants underwent therapy during the four years, which could have had a positive impact on their work habits and aided in overcoming work addiction.

Several clinical and practical implications can be drawn from the results. For women, work addiction appears to be a more stable issue over time, with socially prescribed perfectionism as the primary predictor of chronicity. For men, high self-expectations and psychological distress also emerge as risk factors. Therefore, if maintaining employees’ occupational health is among their core priorities, organizations should avoid promoting excessive perfectionism and setting unrealistically high expectations for their employees. Doing so can elevate the risk of work addiction, which might initially appear advantageous to companies but carries numerous costs and negative consequences for the organization. Research has demonstrated that work addiction correlates with higher rates of burnout, increased physical and mental health problems, elevated absenteeism, and a greater intention to quit [2, 11, 111]. Furthermore, it is crucial for companies to encourage employees to find intrinsic motivation in their work, as it is an independent factor in work addiction. Conversely, reinforcing extrinsic material regulation may heighten the risk of work addiction, as well as the significance of work, whereas motivations emphasizing enjoyment are less likely to do so. Workplace organizations must prioritize the mental health of their employees and even screen for symptoms such as anxiety or depression. The presence of these symptoms, especially in men, increases the risk of work addiction. Elevated workplace stress contributes to anxiety and depressive symptoms [112], so efforts to minimize stress can also reduce the risk of work addiction. Finally, more general proposals for the prevention and treatment of work addiction can be suggested. It is valuable to assist individuals in reducing maladaptive perfectionist thoughts and rumination. Several types of interventions have proven effective for this, such as Cognitive Behavioral Therapy (CBT) and mindfulness-based therapies [113, 114]. While there is very limited data on the effective treatment of work addiction, there is evidence supporting the effectiveness of meditation-based interventions [115]. It may be hypothesized that employing CBT and mindfulness therapy for work addiction could be beneficial, making perfectionism, negative emotions, and rumination central to the therapeutic process. Additionally, greater attention should be given to the treatment of female individuals with work addiction, who are not only more numerous, at least in Western culture, but also appear to be more susceptible to work addiction. Addressing their specific needs could be crucial in promoting change and recovery in our society.

While our research was conducted using a longitudinal design, it is important to acknowledge several limitations. (i) We employed convenience sampling, limiting the generalizability of our findings to the broader population. (ii) Notably, a substantial number of individuals declined to participate in the second data collection, potentially introducing bias into the results. Analysis of the data shows that those who dropped out before the second data collection tended to have higher self-esteem, along with elevated self-oriented and socially prescribed perfectionism. Consequently, it is possible that many individuals affected by work addiction did not continue with the research. (iii) Another limitation pertains to our use of self-report questionnaires, which may introduce biases associated with social desirability, self-knowledge, and memory processes. An alternative to self-report questionnaires could involve the use of other evaluation methods, such as multi-rater techniques, which would allow for a more accurate assessment by incorporating different perspectives. This could help mitigate potential biases related to self-evaluation. (iv) Additionally, when we analyzed data based on a cut-off value, only 37 people (6.31%) were included in the increased work addiction group, resulting in a significantly smaller sample size compared to the other four groups. This discrepancy could introduce biases in comparative analyses. (v) Likewise, in the separate latent class analysis conducted for each sex, especially for men (resulting in five obtained groups), the sample sizes were small, which could potentially introduce bias into the results. When sample sizes are small, the estimation of class membership and the relationships between covariates and latent classes may be less stable and more prone to variability. This could lead to overfitting or unreliable class assignments, which might skew the results. Therefore, these findings should be interpreted with caution, as the small sample sizes could limit the robustness and generalizability of the conclusions. (vi) In the present study, the relatively small sample sizes in both gender groups precluded the possibility of validating the clusters using a splitting procedure. Specifically, random splitting of the dataset into two equal groups—one for constructing the LCA model and the other for testing model stability in terms of the number of classes, classification accuracy, and posterior predictive probabilities—was not feasible. Therefore, validation will require a separate sample. (vii) During multinomial regression analysis, we attempted to examine the variable’s impact by entering too many predictors simultaneously. This approach may not be suitable for groups with a limited number of elements. (viii) It is important to highlight that our current research predominantly focused on individual dimensions, while numerous situational, environmental, and workplace factors —such as job demands [116], lower job resources [117], competitive climate [87], managerial support [118], job control [119], and coworkers’ work addiction [120]—also contribute to work addiction. (ix) Another limitation of our study is that, although we conducted a longitudinal analysis, data were collected at only two time points. (x) In our longitudinal analysis, we examined one type of temporal relationship between variables; however, this does not rule out the relevance of analyzing it in the opposite direction (i.e., the effect of work addiction on subsequent work motivations). It is possible that the relationships between the variables would also be supported in this alternative model. Future research should consider testing both temporal relationships simultaneously. (xi) Finally, it is also important to emphasize that during the 4-year period between the two data collection dates, the Covid-19 pandemic occurred. We originally planned a two-year interval to assess the stability of work addiction. However, due to the COVID-19 pandemic, data collection in 2020 and 2021 was postponed, as work-related conditions were highly disrupted. The second wave was ultimately conducted in 2022, once the situation in Hungary had stabilized.

## Conclusions

Our research was specifically designed to address a critical gap in the existing knowledge by investigating the individual factors that contribute to the persistence of chronic work addiction. To this end, we employed a person-centered approach, which enabled us to identify distinct subgroups based on longitudinal symptom patterns and examine how individual psychological characteristics relate to these trajectories. Our longitudinal research has revealed that personality traits have an impact on the persistence of work addiction. Individuals who perceive themselves as less valuable, constantly strive to meet others’ needs, have more emotional disturbances, and find personal significance in their work without experiencing enjoyment are at a greater risk of work addiction. As work addiction is a maladaptive phenomenon with numerous negative consequences, its prevention and treatment are of paramount importance. To effectively address chronic work addiction, it is imperative to place greater emphasis on the individual risk factors we have identified.

## Electronic supplementary material

Below is the link to the electronic supplementary material.

Supplementary Material 1

## Data Availability

All data and code for the analyses have been deposited in the Open Science Framework page and they are available at: https://osf.io/fkupx/?view_only=787c4c07428241d488a08a737b42291a

## Declarations

### Abbreviations

All abbreviations are explained in the paper at the time of their first occurrence.

## References

[1] Tóth-Király I, Bőthe B, Orosz G. Seeing the forest through different trees: A social psychological perspective of work addiction: commentary on: ten Myths about work addiction (Griffiths et al., 2018). J Behav Addict. 2018;7(4):875–9. 30556783 10.1556/2006.7.2018.122 PMC6376368

[2] Clark MA, Michel JS, Zhdanova L, Pui SY, Baltes BB. All work and no play? A meta-analytic examination of the correlates and outcomes of workaholism. J Manag. 2016;42(7):1836–73.

[3] Andreassen CS, Griffiths MD, Hetland J, Kravina L, Jensen F, Pallesen S. The prevalence of workaholism: A survey study in a nationally representative sample of Norwegian employees. PLoS ONE. 2014;9(8):e102446. 25118877 10.1371/journal.pone.0102446 PMC4131865

[4] van Berk B, Ebner C, Rohrbach-Schmidt D. Wer hat Nie Richtig Feierabend? Eine analyse Zur verbreitung von suchthaftem arbeiten in Deutschland. Arbeit. 2022;31(3):257–82.

[5] Kang S. Workaholism in Korea: prevalence and Socio-Demographic differences. Front Psychol. 2020;11:569744. 33424681 10.3389/fpsyg.2020.569744 PMC7786266

[6] Urbán R, Kun B, Mózes T, Soltész P, Paksi B, Farkas J, et al. A Four-Factor model of work addiction: the development of the work addiction risk test revised. Eur Addict Res. 2019;25(3):145–60. 30982051 10.1159/000499672

[7] Atroszko PA, Demetrovics Z, Griffiths MD. Beyond the Myths about work addiction: toward a consensus on definition and trajectories for future studies on problematic overworking. J Behav Addict. 2019;8:7–15. 30920291 10.1556/2006.8.2019.11 PMC7044606

[8] Bartczak M, Ogińska-Bulik N. Workaholism and mental health among Polish academic workers. Int J Occup Saf Ergon JOSE. 2012;18(1):3–13. 22429525 10.1080/10803548.2012.11076910

[9] Lanzo L, Aziz S, Wuensch K. Workaholism and incivility: stress and psychological capital’s role. Int J Workplace Health Manag. 2016;9:165–83.

[10] Yang X, Qiu D, Lau MCM, Lau JTF. The mediation role of work-life balance stress and chronic fatigue in the relationship between workaholism and depression among Chinese male workers in Hong Kong. J Behav Addict. 2020;9(2):483–90. 32663383 10.1556/2006.2020.00026 PMC8939414

[11] Matsudaira K, Shimazu A, Fujii T, Kubota K, Sawada T, Kikuchi N, et al. Workaholism as a risk factor for depressive mood, disabling back pain, and sickness absence. PLoS ONE. 2013;8(9):e75140. 24086457 10.1371/journal.pone.0075140 PMC3783450

[12] Salanova M, López-González AA, Llorens S, del Líbano M, Vicente-Herrero MT, Tomás-Salvá M. Your work May be killing you! Workaholism, sleep problems and cardiovascular risk. Work Stress. 2016;30(3):228–42.

[13] Kun B, Fetahu D, Mervó B, Magi A, Eisinger A, Paksi B, et al. Work addiction and stimulant use: latent profile analysis in a representative population study. Int J Ment Health Addict. 2023;23:1–22.

[14] Kun B, Paksi B, Eisinger A, Kökönyei G, Demetrovics Z. Driving and mobile phone use: work addiction predicts hazardous but not excessive mobile phone use in a longitudinal study of young adults. J Behav Addict. 2024;13(1):66–75. 38459979 10.1556/2006.2024.00007 PMC10988412

[15] Bakker AB, Demerouti E, Burke R. Workaholism and relationship quality: A spillover-crossover perspective. J Occup Health Psychol. 2009;14:23–33. 19210044 10.1037/a0013290

[16] Kenyhercz V, Mervó B, Lehel N, Demetrovics Z, Kun B. Work addiction and social functioning: A systematic review and five meta-analyses. PLoS ONE. 2024;19(6):e0303563. 38833505 10.1371/journal.pone.0303563 PMC11149883

[17] Torp S, Lysfjord L, Midje HH. Workaholism and work–family conflict among university academics. High Educ. 2018;76(6):1071–90.

[18] Robinson BE, Flowers C, Carroll J. Work stress and marriage: A theoretical model examining the relationship between workaholism and marital cohesion. Int J Stress Manag. 2001;8(2):165–75.

[19] Kravina L, Falco A, Girardi D, De Carlo NA. Workaholism among management and workers in an Italian cooperative enterprise. TPM-Test Psychom Methodol Appl Psychol. 2010;17:201–16.

[20] Schaufeli WB, Bakker AB, van der Heijden FMMA, Prins JT. Workaholism, burnout and well-being among junior Doctors: the mediating role of role conflict. Work Stress. 2009;23(2):155–72.

[21] Andreassen SC, Ursin H, Eriksen HR. The relationship between strong motivation to work, workaholism, and health. Psychol Health. 2007;22(5):615–29.

[22] Burke RJ. Workaholism components, job satisfaction, and career progress. J Appl Soc Psychol. 2001;31:2339–56.

[23] Griffiths MD, Karanika-Murray M. Contextualising over-engagement in work: towards a more global Understanding of workaholism as an addiction. J Behav Addict. 2012;1(3):87–95. 26165458 10.1556/JBA.1.2012.002

[24] Ng TWH, Sorensen KL, Feldman DC. Dimensions, antecedents, and consequences of workaholism: A conceptual integration and extension. J Organ Behav. 2007;28(1):111–36.

[25] Oates WE. Confessions of a workaholic: the facts about work addiction. New York: World Pub. Co.; 1971.

[26] Robinson BE. Chained to the desk: A guidebook for workaholics, their partners and children, and the clinicians who treat them, 2nd ed. New York, NY, US: New York University Press; 2007. xiv, 274 p. (Chained to the desk: A guidebook for workaholics, their partners and children, and the clinicians who treat them, 2nd ed).

[27] Halvorson MA, Lengua LJ, Smith GT, King KM. Pathways of personality and learning risk for addictive behaviors: A systematic review of mediational research on the acquired preparedness model. J Pers. 2023;91(3):613–37. 35900782 10.1111/jopy.12761 PMC10351414

[28] Zilberman N, Yadid G, Efrati Y, Neumark Y, Rassovsky Y. Personality profiles of substance and behavioral addictions. Addict Behav. 2018;82:174–81. 29547799 10.1016/j.addbeh.2018.03.007

[29] Kun B, Takacs ZK, Richman MJ, Griffiths MD, Demetrovics Z. Work addiction and personality: A meta-analytic study. J Behav Addict. 2020;9(4):945–66. 33361486 10.1556/2006.2020.00097 PMC8969726

[30] Andreassen CS, Bjorvatn B, Moen BE, Waage S, Magerøy N, Pallesen S. A longitudinal study of the relationship between the five-factor model of personality and workaholism. TPM-Test Psychom Methodol Appl Psychol. 2016;23:285–98.

[31] Atroszko PA, Andreassen CS, Griffiths MD, Pallesen S. The relationship between study addiction and work addiction: A cross-cultural longitudinal study. J Behav Addict. 2016;5(4):708–14. 27842448 10.1556/2006.5.2016.076 PMC5370377

[32] Falco A, Girardi D, Corso LD, De Carlo A, Di Sipio A. Does workload moderate the association between perfectionism and workaholism?? J Pers Psychol. 2020;19(4):164–73.

[33] Nolen-Hoeksema S, Wisco BE, Lyubomirsky S. Rethinking rumination. Perspect Psychol Sci J Assoc Psychol Sci. 2008;3(5):400–24. 10.1111/j.1745-6924.2008.00088.x 26158958

[34] Nolen-Hoeksema S. Responses to depression and their effects on the duration of depressive episodes. J Abnorm Psychol. 1991;100(4):569–82. 1757671 10.1037//0021-843x.100.4.569

[35] Aziz S, Zamary S, Wuensch K. The endless pursuit for self-validation through attainment: an examination of self-esteem in relation to workaholism. Personal Individ Differ. 2018;121:74–9.

[36] Serrano-Fernández MJ, Boada-Grau J, Boada-Cuerva M, Vigil-Colet A. Work addiction as a predictor of anxiety and depression. Work Read Mass. 2021;68(3):779–88. 10.3233/WOR-203411 33612520

[37] Kun B, Urbán R, Bőthe B, Griffiths MD, Demetrovics Z, Kökönyei G. Maladaptive rumination mediates the relationship between Self-Esteem, perfectionism, and work addiction: A largescale survey study. Int J Environ Res Public Health. 2020;17(19):7332. 33049921 10.3390/ijerph17197332 PMC7579015

[38] McCrae RR, Costa Jr. PT. Personality trait structure as a human universal. Am Psychol. 1997;52(5):509–16. 9145021 10.1037//0003-066x.52.5.509

[39] Bäcklund C, Elbe P, Gavelin HM, Sörman DE, Ljungberg JK. Gaming motivations and gaming disorder symptoms: A systematic review and meta-analysis. J Behav Addict. 2022;11(3):667–88. 36094861 10.1556/2006.2022.00053 PMC9872536

[40] Cooper ML. Motivations for alcohol use among adolescents: development and validation of a four-factor model. Psychol Assess. 1994;6(2):117–28.

[41] Hagfors H, Castrén S, Salonen AH. How gambling motives are associated with socio-demographics and gambling behavior - A Finnish population study. J Behav Addict. 2022;11(1):63–74. 35275094 10.1556/2006.2022.00003 PMC9109631

[42] Mosier SK, Workaholics. An analysis of their stress, success and priorities. University of Texas at Austin; 1983.

[43] Buelens M, Poelmans SAY. Enriching the spence and Robbins’ typology of workaholism: demographic, motivational and organizational correlates. J Organ Change Manag. 2004;17(5):440–58.

[44] Van Beek I, Taris TW, Schaufeli WB. Workaholic and work engaged employees: dead ringers or worlds apart? J Occup Health Psychol. 2011;16(4):468–82. 21787085 10.1037/a0024392

[45] Taris TW, van Beek I, Schaufeli WB. Why do perfectionists have a higher burnout risk than others? The mediational effect of workaholism. Rom J Appl Psychol. 12(1):1–7.

[46] Scott KS, Moore KS, Miceli MP. An exploration of the meaning and consequences of workaholism. Hum Relat. 1997;50(3):287–314.

[47] Spence JT, Robbins AS. Workaholism: definition, measurement, and preliminary results. J Pers Assess. 1992;58(1):160–78. 16370875 10.1207/s15327752jpa5801_15

[48] Deci EL, Ryan RM. Self-determination theory. Handbook of theories of social psychology. Volume 1. Thousand Oaks, CA: Sage Publications Ltd; 2012. pp. 416–36.

[49] Ryan RM, Deci EL. Self-determination theory and the facilitation of intrinsic motivation, social development, and well-being. Am Psychol. 2000;55(1):68–78. 11392867 10.1037//0003-066x.55.1.68

[50] Van den Broeck A, Schreurs B, De Witte H, Vansteenkiste M, Germeys F, Schaufeli W. Understanding workaholics’ motivations: A Self-Determination perspective. Appl Psychol. 2011;60(4):600–21.

[51] Stoeber J, Davis CR, Townley J. Perfectionism and workaholism in employees: the role of work motivation. Personal Individ Differ. 2013;55(7):733–8.

[52] van Beek I, Hu Q, Schaufeli WB, Taris TW, Schreurs BHJ. For fun, love, or money: what drives workaholic, engaged, and burned-out employees at work? Appl Psychol Int Rev. 2012;61(1):30–55.

[53] Taris TW, van Beek I, Schaufeli WB. The motivational Make-Up of workaholism and work engagement: A longitudinal study on need satisfaction, motivation, and heavy work investment. Front Psychol. 2020;11:1419. 32714248 10.3389/fpsyg.2020.01419 PMC7344159

[54] Anderson KG, Briggs KEL, White HR. Motives to drink or not to drink: longitudinal relations among personality, motives, and alcohol use across adolescence and early adulthood. Alcohol Clin Exp Res. 2013;37(5):860–7. 23278843 10.1111/acer.12030 PMC3620892

[55] Hagfors H, Vuorinen I, Savolainen I, Oksanen A. A longitudinal study of gambling motives, problem gambling and need frustration. Addict Behav. 2023;144:107733. 37119715 10.1016/j.addbeh.2023.107733

[56] Chang Smei, Lin SSJ. Online gaming motive profiles in late adolescence and the related longitudinal development of stress, depression, and problematic internet use. Comput Educ. 2019;135(1):123–37.

[57] Stevens MW, Dorstyn D, Delfabbro PH, King DL. Global prevalence of gaming disorder: A systematic review and meta-analysis. Aust N Z J Psychiatry. 2021;55(6):553–68. 33028074 10.1177/0004867420962851

[58] Maraz A, van den Brink W, Demetrovics Z. Prevalence and construct validity of compulsive buying disorder in shopping mall visitors. Psychiatry Res. 2015;228(3):918–24. 26027442 10.1016/j.psychres.2015.04.012

[59] Grijalva E, Newman DA, Tay L, Donnellan MB, Harms PD, Robins RW, et al. Gender differences in narcissism: A meta-analytic review. Psychol Bull. 2015;141(2):261–310. 25546498 10.1037/a0038231

[60] Kling KC, Hyde JS, Showers CJ, Buswell BN. Gender differences in self-esteem: a meta-analysis. Psychol Bull. 1999;125(4):470–500. 10414226 10.1037/0033-2909.125.4.470

[61] Beiler-May A, Williamson RL, Clark MA, Carter NT. Gender Bias in the measurement of workaholism. J Pers Assess. 2017;99(1):104–10. 27409147 10.1080/00223891.2016.1198795

[62] Robinson BE, Post P, Khakee JF. Test-retest reliability of the work addiction risk test. Percept Mot Skills. 1992;74(3):926. 1608730 10.2466/pms.1992.74.3.926

[63] Falco A, Girardi D, De Carlo A, Andreassen CS, Dal Corso L. Work addiction among bank employees in Italy: A contribution to validation of the Bergen work addiction scale with a focus on measurement invariance across gender and managerial status. Sustainability. 2022;14(21):13714.

[64] Buono C, Spagnoli P, Clark M, Haynes NJ, Molinaro D, Balducci C. A further examination of the multidimensional workaholism scale (MWS) in Italy and U.S: measurement equivalence, convergent, discriminant, and predictive validity. J Pers Assess. 2024;106(3):384–95. 38010899 10.1080/00223891.2023.2276268

[65] Kun B, Magi A, Felvinczi K, Demetrovics Z, Paksi B. [Prevalence, sociodemographic, and psychological characteristics of work addiction in the Hungarian adult population: results of a nationally representative survey]. Psychiatr Hung. 2020;35(3):289–306. 32643619

[66] Andreassen CS, Griffiths MD, Hetland J, Pallesen S. Development of a work addiction scale. Scand J Psychol. 2012;53(3):265–72. 22490005 10.1111/j.1467-9450.2012.00947.x

[67] Ormel J, Wohlfarth T. How neuroticism, long-term difficulties, and life situation change influence psychological distress: A longitudinal model. J Pers Soc Psychol. 1991;60(5):744–55. 2072254 10.1037//0022-3514.60.5.744

[68] Orosz G, Dombi E, Andreassen CS, Griffiths MD, Demetrovics Z. Analyzing models of work addiction: single factor and bi-factor models of the Bergen work addiction scale. Int J Ment Health Addict. 2016;14:662–71.

[69] Griffiths M. A components model of addiction within a biopsychosocial framework. J Subst Use. 2005;10(4):191–7.

[70] Urbán R, Szigeti R, Kökönyei G, Demetrovics Z. Global self-esteem and method effects: competing factor structures, longitudinal invariance and response styles in adolescents. Behav Res Methods. 2014;46(2):488–98. 24061931 10.3758/s13428-013-0391-5 PMC3947716

[71] Rosenberg M. Society and the adolescent Self-Image. Princeton University Press; 1965.

[72] Cox BJ, Enns MW, Clara IP. The multidimensional structure of perfectionism in clinically distressed and college student samples. Psychol Assess. 2002;14(3):365–73. 12214443

[73] Hewitt PL, Flett GL. Perfectionism and depression: A multidimensional analysis. J Soc Behav Personal. 1990;5(5):423–38.

[74] Juwono ID, Kun B, Demetrovics Z, Urbán R. Healthy and unhealthy dimensions of perfectionism: perfectionism and mental health in Hungarian adults. Int J Ment Health Addict. 2023;21(5):3017–32.

[75] Leckelt M, Wetzel E, Gerlach TM, Ackerman RA, Miller JD, Chopik WJ, et al. Validation of the narcissistic admiration and rivalry questionnaire short scale (NARQ-S) in convenience and representative samples. Psychol Assess. 2018;30(1):86–96. 28252974 10.1037/pas0000433

[76] Derogatis LR, Fitzpatrick M. The SCL-90-R, the brief symptom inventory (BSI), and the BSI-18. The use of psychological testing for treatment planning and outcomes assessment: instruments for adults. Volume 3, 3rd ed. Mahwah, NJ, US: Lawrence Erlbaum Associates; 2004. pp. 1–41.

[77] Urbán R, Kun B, Farkas J, Paksi B, Kökönyei G, Unoka Z, et al. Bifactor structural model of symptom checklists: SCL-90-R and brief symptom inventory (BSI) in a non-clinical community sample. Psychiatry Res. 2014;216(1):146–54. 24524946 10.1016/j.psychres.2014.01.027

[78] Treynor W, Gonzalez R, Nolen-Hoeksema S. Rumination reconsidered: A psychometric analysis. Cogn Ther Res. 2003;27(3):247–59.

[79] Eszlári N, Kökönyei G. Ruminatív Válaszstílus Kérdőív (Ruminative response scale, RRS). In: Horváth Z, Urbán R, Kökönyei G, Demetrovics Z, editors. Kérdőíves Módszerek a klinikai És Egészségpszichológiai Kutatásban És Gyakorlatban I. Budapest: Medicina; 2021. pp. 127–32.

[80] Gagné M, Forest J, Vansteenkiste M, Crevier-Braud L, van den Broeck A, Aspeli AK, et al. The multidimensional work motivation scale: validation evidence in seven languages and nine countries. Eur J Work Organ Psychol. 2015;24(2):178–96.

[81] Deci EL, Ryan RM. Intrinsic motivation and Self-Determination in human behavior. Handbook of theories of social psychology. Volume 1. Thousand Oaks, CA: Sage Publications Ltd; 2012. pp. 416–36.

[82] Kun B, Martos T, Bőthe B, Tóth-Király I. Többdimenziós munkamotiváció Skála: (Multidimensional work motivation scale, MWMS). In: Horváth Z, Urbán R, Kökönyei G, Demetrovics Z, editors. Kérdőíves Módszerek a klinikai És Egészségpszichológiai Kutatásban És Gyakorlatban I. Budapest: Medicina; 2021. pp. 492–6.

[83] Clark S, Muthén B. Relating Latent Class Analysis Results to Variables not Included in the Analysis. 2009.

[84] Asparouhov T, Muthen B. Auxiliary Variables in Mixture Modeling: Using the BCH Method in Mplus to Estimate a Distal Outcome Model and an Arbitrary Secondary Model.:22.

[85] Muthén LK, Muthen B. Mplus user’s guide: statistical analysis with latent variables, user’s guide. Muthén & Muthén; 2017.

[86] Griffiths MD, Demetrovics Z, Atroszko PA. Ten Myths about work addiction. J Behav Addict. 2018;7(4):845–57. 29409339 10.1556/2006.7.2018.05 PMC6376361

[87] Keller AC, Spurk D, Baumeler F, Hirschi A. Competitive climate and workaholism: negative sides of future orientation and calling. Personal Individ Differ. 2016;96:122–6.

[88] Schaufeli WB. Heavy work investment, personality and organizational climate. J Manag Psychol. 2016;31(6):1057–73.

[89] Egan SJ, Wade TD, Shafran R. Perfectionism as a transdiagnostic process: a clinical review. Clin Psychol Rev. 2011;(2):203–12. 10.1016/j.cpr.2010.04.009 20488598

[90] Flett GL, Hewitt PL, Nepon T, Sherry SB, Smith M. The destructiveness and public health significance of socially prescribed perfectionism: A review, analysis, and conceptual extension. Clin Psychol Rev. 2022;93:102130. 35216826 10.1016/j.cpr.2022.102130

[91] van Wijhe C, Peeters M, Schaufeli W, van den Hout M. Understanding workaholism and work engagement: the role of mood and stop rules. Career Dev Int. 2011;16(3):254–70.

[92] Johnson DP, Whisman MA. Gender differences in rumination: A meta-analysis. Personal Individ Differ. 2013;55(4):367–74. 10.1016/j.paid.2013.03.019 PMC3786159 24089583

[93] Sand L, Bøe T, Shafran R, Stormark KM, Hysing M. Perfectionism in adolescence: associations with gender, age, and socioeconomic status in a Norwegian sample. Front Public Health. 2021;25(9):688811. 10.3389/fpubh.2021.688811 PMC8424040 34513782

[94] Weidmann R, Chopik WJ, Ackerman RA, Allroggen M, Bianchi EC, Brecheen C, et al. Age and gender differences in narcissism: A comprehensive study across eight measures and over 250,000 participants. J Pers Soc Psychol. 2023;124(6):1277–98. 37184962 10.1037/pspp0000463 PMC10188200

[95] Milosevic A, Ledgerwood DM. The subtyping of pathological gambling: A comprehensive review. Clin Psychol Rev. 2010;30(8):988–98. 20655134 10.1016/j.cpr.2010.06.013

[96] Wang HY, Cheng C. The associations between gaming motivation and internet gaming disorder: systematic review and Meta-analysis. JMIR Ment Health. 2022;9(2):e23700. 35175204 10.2196/23700 PMC8895288

[97] McCarthy DM, Tomlinson KL, Anderson KG, Marlatt GA, Brown SA. Relapse in Alcohol- and Drug-Disordered adolescents with comorbid psychopathology: changes in psychiatric symptoms. Psychol Addict Behav. 2005;19(1):28–34. 15783275 10.1037/0893-164X.19.1.28

[98] Keller AC, Meier LL, Gross S, Semmer NK. Gender differences in the association of a high quality job and self-esteem over time: A multiwave study. Eur J Work Organ Psychol. 2015;24(1):113–25.

[99] Pilar de Luis Carnicer M, Martínez Sánchez A, Pérez Pérez M. José Vela Jiménez M. Gender differences of mobility: analysis of job and work-family factors. Women Manag Rev. 2003;18(4):199–219.

[100] Söderberg M, Härenstam A, Rosengren A, Schiöler L, Olin AC, Lissner L, et al. Psychosocial work environment, job mobility and gender differences in turnover behaviour: a prospective study among the Swedish general population. BMC Public Health. 2014;14:605. 24927628 10.1186/1471-2458-14-605 PMC4073185

[101] Richardsen AM, Traavik LEM, Burke RJ. Women and work stress: more and different?? In: Connerley M, Wu J, editors. Handbook on Well-Being of working women. International handbooks of Quality-of-Life. Dordrecht: Springer; 2016.

[102] Bönte W. Gender differences in competitive preferences: new cross-country empirical evidence. Appl Econ Lett. 2015;22(1):71–5.

[103] Huikku J, Myllymäki ER, Ojala H. Gender differences in the first course in accounting: an achievement goal approach. Br Acc Rev. 2022;54(3):101081.

[104] Kaur N, Puria A, Kumar A, Chaudhury S, Goyal E, Singh VP. Caregiver burden among working women and homemakers taking care of psychiatric patients. Ind Psychiatry J. 2021;30(Suppl 1):S166–71. 34908684 10.4103/0972-6748.328809 PMC8611542

[105] Stephens MA, Franks MM, Townsend AL. Stress and rewards in women’s multiple roles: the case of women in the middle. Psychol Aging. 1994;9(1):45–52. 8185867 10.1037//0882-7974.9.1.45

[106] Taparia M, Lenka U. An integrated conceptual framework of the glass ceiling effect. J Organ Eff People Perform. 2022;9(3):372–400.

[107] De Clercq D, Brieger SA. When discrimination is worse, autonomy is key: how women entrepreneurs leverage job autonomy resources to find Work–Life balance. J Bus Ethics. 2022;177(3):665–82.

[108] Mariezcurrena R. Recovery from addictions without treatment: literature review. Scand J Behav Ther. 1994;23(3–4):131–54.

[109] Smart RG. Spontaneous recovery in alcoholics: A review and analysis of the available research. Drug Alcohol Depend. 1976;1(4):277–85. 797563 10.1016/0376-8716(76)90023-5

[110] Toneatto T, Cunningham J, Hodgins D, Adams M, Turner N, Koski-Jannes A. Recovery from problem gambling without formal treatment. Addict Res Theory. 2008;16(2):111–20.

[111] Van Beek I, Taris W, Schaufeli TB, Brenninkmeijer W. Heavy work investment: its motivational make-up and outcomes. J Manag Psychol. 2013;29(1):46–62.

[112] Melchior M, Caspi A, Milne BJ, Danese A, Poulton R, Moffitt TE. Work stress precipitates depression and anxiety in young, working women and men. Psychol Med. 2007;37(8):1119–29. 17407618 10.1017/S0033291707000414 PMC2062493

[113] Galloway R, Watson H, Greene D, Shafran R, Egan SJ. The efficacy of randomised controlled trials of cognitive behaviour therapy for perfectionism: a systematic review and meta-analysis. Cogn Behav Ther. 2022;51(2):170–84. 34346282 10.1080/16506073.2021.1952302

[114] James K, Rimes KA. Mindfulness-Based cognitive therapy versus pure cognitive behavioural Self-Help for perfectionism: a pilot randomised study. Mindfulness. 2018;9(3):801–14. 29875882 10.1007/s12671-017-0817-8 PMC5968046

[115] Gordon WV, Shonin E, Dunn TJ, Garcia-Campayo J, Demarzo MMP, Griffiths MD. Meditation awareness training for the treatment of workaholism: A controlled trial. J Behav Addict. 2017;6(2):212–20. 28425778 10.1556/2006.6.2017.021 PMC5520118

[116] Andreassen CS, Nielsen MB, Pallesen S, Gjerstad J. The relationship between psychosocial work variables and workaholism: findings from a nationally representative survey. Int J Stress Manag. 2019;26(1):1–10.

[117] Molino M, Bakker AB, Ghislieri C. The role of workaholism in the job demands-resources model. Anxiety Stress Coping. 2016;(4):400–14. 10.1080/10615806.2015.1070833 26168061

[118] Mazzetti G, Vignoli M, Schaufeli WB, Guglielmi D. Work addiction and presenteeism: the buffering role of managerial support. Int J Psychol J Int Psychol. 2019;54(2):174–9. 10.1002/ijop.12449 28791675

[119] Schaufeli WB, Taris TW, Bakker AB. It takes two to Tango: workaholism is working excessively and working compulsively. In: Burke RJ, Cooper CL, editors. The long work hours culture: causes, consequences and choices. Bingly, UK: Emerald; 2008.

[120] Atroszko PA, Kun B, Buźniak A, Czerwiński SK, Schneider Z, Woropay-Hordziejewicz N, et al. Perceived coworkers’ work addiction: scale development and associations with one’s own workaholism, job stress, and job satisfaction in 85 cultures. J Behav Addict. 2025;14(1):246–62. 40014059 10.1556/2006.2025.00011 PMC11974416

