# Supplementary material for: Long-term chronicity of work addiction: the role of personality and work motivations
Source: BMC Psychol. 2025 May 29;13:578. doi: 10.1186/s40359-025-02907-6 (PMC12124100; doi:10.1186/s40359-025-02907-6)
Supplement: Supplementary file 1 — Supplementary Material 1 [file 40359_2025_2907_MOESM1_ESM.docx]

**Supplementary Materials**

**Table S1**

*Descriptive Statistics of the Variables and Correlations Between Them*

|  |  | 1 | 2 | 3 | 4 | 5 | 6 | 7 | 8 | 9 | 10 | 11 | 12 | 13 | 14 | 15 | 16 | 17 |
| --- | --- | --- | --- | --- | --- | --- | --- | --- | --- | --- | --- | --- | --- | --- | --- | --- | --- | --- |
| 1 | BWAS T1 |  |  |  |  |  |  |  |  |  |  |  |  |  |  |  |  |  |
| 2 | BWAS T2 | .603** |  |  |  |  |  |  |  |  |  |  |  |  |  |  |  |  |
| 3 | RSES | –.326** | –.254** |  |  |  |  |  |  |  |  |  |  |  |  |  |  |  |
| 4 | SOP T1 | .400** | .270** | –.078 |  |  |  |  |  |  |  |  |  |  |  |  |  |  |
| 5 | OOP T1 | .194** | .093* | .080 | .346** |  |  |  |  |  |  |  |  |  |  |  |  |  |
| 6 | SPP T1 | .382** | .339** | –.448** | .360** | .030 |  |  |  |  |  |  |  |  |  |  |  |  |
| 7 | NARQ–S Admiration T1 | .043 | .017 | .385** | .250** | .239** | –.008 |  |  |  |  |  |  |  |  |  |  |  |
| 8 | NARQ–S  Rivalry T1 | .157** | .162** | –.181** | .255** | .231** | .251** | .374** |  |  |  |  |  |  |  |  |  |  |
| 9 | PD T1 | .469** | .348** | –698** | .179** | –.006 | .484** | –130** | 340** |  |  |  |  |  |  |  |  |  |
| 10 | RRS  Brooding T1 | .357** | .285** | –.548** | .216** | –.024 | .446** | –.280 | .267** | .679** |  |  |  |  |  |  |  |  |
| 11 | RRS Reflective Pondering T1 | .254** | .205** | –.207** | .174** | .003 | .242** | .104* | .088* | .363** | .433** |  |  |  |  |  |  |  |
| 12 | MWMS Amotivation T1 | –.080 | –.066 | –.238** | –.102* | –.117** | .110** | –.015 | .286** | .296** | .201** | .120** |  |  |  |  |  |  |
| 13 | MWMS Extrinsic Social Regulation T1 | .285** | .201** | –.261** | .322** | .112** | .396** | .154** | .276** | .345** | .343** | .119** | .081 |  |  |  |  |  |
| 14 | MWMS Extrinsic Material Regulation T1 | .190** | .147** | –.200** | .165** | .026 | .322** | .094* | .238** | .299** | .259** | .064 | .086* | .524** |  |  |  |  |
| 15 | MWMS Introjected Regulation T1 | .376** | .261** | –.145** | .479** | .254** | .231** | .176** | .108** | .190** | .224** | .200** | –.256** | .407* | .164** |  |  |  |
| 16 | MWMS Identified Regulation T1 | .325** | .199** | .122** | .438** | .252** | .016 | .148** | –.103* | –0.83* | –.014 | –0.76 | –.504** | .083* | –.051 | .605** |  |  |
| 17 | MWMS  Intrinsic Motivation T1 | .056 | .043 | .310** | .117** | .093* | –.094* | .133** | –.218** | –.319** | –.166** | –.002 | –.657** | –.075 | –.134** | .340** | .548** |  |
| Range | | 7-35 | 7-35 | 10-40 | 5-35 | 5-35 | 5-35 | 3-18 | 3-18 | 12-48 | 5-20 | 5-20 | 3-21 | 3-21 | 3-21 | 4-28 | 3-21 | 3-21 |
| *M* (*SD*) | | 21.51  (5.16) | 19.55  (5.24) | 28.82  (5.45) | 26.19  (6.19) | 20.39  (6.08) | 16.62  (6.84) | 10.49  3.46 | 8.75  (3.19) | 23.85  (7.02) | 10.41  (3.19) | 10.81  (3.26) | 6.71  (4.59) | 10.64  (4.84) | 10.98  (5.11) | 18.78  (6.11) | 16.59  (4.02) | 14.20  (5.23) |
| Cronbach’s α | | .800 | .810 | .882 | .867 | .794 | .791 | .768 | .577 | .893 | .749 | .766 | .885 | .852 | .803 | .840 | .914 | .954 |

*Note*. BWAS, Bergen Work Addiction Scale, RSES; Rosenberg Self-Esteem Scale; SOP, Self-Oriented Perfectionism; OOP, Other-Oriented Perfectionism; SPP, Socially Prescribed Perfectionism; NARQ-S, Narcissistic Admiration and Rivalry Questionnaire Short scale; PD, psychopathological distress; RRS, Ruminative Response Scale; MWMS, Multidimensional Work Motivation Scale; *M*, mean; *SD*, standard deviation.

* *p* < .05; ** *p* < .01

**Table S2**

*Socio-Demographic Characteristics and Variable Means for Dropouts and Participants Who Remained.*

|  | | Dropouts  (*N* = 1,157) | Final respondents  (*N* = 586) | Difference |
| --- | --- | --- | --- | --- |
| **Mean age (*SD*)** | | **36.34 (9.33)** | **37.97 (9.16)** | ***t*(df) = –3.47 (1741); *p* < .001** |
| *N* (%) of males | | 587 (50.7) | 285 (48.6) | *χ^2^*(df) = 0.69 (1); *p* = .408 |
| **Level of education**  ***N* (%)** | less than primary level | 0 (0) | 1 (0.2) | ***χ^2^*(df) = 17.72 (5); *p* = .003** |
|  | primary level | 7 (0.6) | 2 (0.3) |  |
|  | vocational | 22 (1.9) | 10 (1.7) |  |
|  | secondary | 293 (25.3) | 102 (17.4) |  |
|  | bachelor’s or master’s degree | 780 (67.4) | 446 (76.1) |  |
|  | doctoral degree | 55 (4.8) | 25 (4.3) |  |
| Marital status  *N* (%) | single | 666 (57.7) | 306 (52.3) | *χ^2^*(df) = 5.094 (3); *p* = .165 |
|  | married | 397 (34.4) | 221 (37.8) |  |
|  | divorced | 86 (7.4) | 55 (9.4) |  |
|  | widow | 6 (0.5) | 3 (0.5) |  |
| Work Addiction | | 21.68 (5.03) | 21.51 (5.16) | *t*(df) = 0.66 (1741); *p* = .511 |
| **Self–Esteem** | | **28.19 (5.57)** | **28.81 (5.45)** | ***t*(df) = –2.23 (1739); *p* = .026** |
| **Self–Oriented Perfectionism** | | **27.15 (5.77)** | **26.19 (6.19)** | ***t*(df) = 3.20 (1738); *p* = .001** |
| Other–Oriented Perfectionism | | 20.21 (6.12) | 20.39 (6.08) | *t(*df) = –0.59 (1739); *p* = .557 |
| **Socially Prescribed Perfectionism** | | **17.92 (6.99)** | **16.62 (6.84)** | ***t*(df) = 3.69 (1740); *p* < .001** |
| Narcissism – Admiration | | 10.79 (3.42) | 10.49 (3.46) | *t*(df) = 1.68 (1740); *p* = .092 |
| Narcissism – Rivalry | | 8.86 (3.25) | 8.75 (3.19) | *t*(df) = 0.65 (1741); *p* = .519 |
| Depression | | 12.37 (3.90) | 12.25 (4.06) | *t*(df) = 0.62 (1741); *p* = .527 |
| Anxiety | | 11.87 (3.59) | 11.61 (3.51) | *t*(df) = 1.42 (1740); *p* = .155 |
| Rumination – Brooding | | 10.66 (3.15) | 10.41 (3.19) | *t*(df) = 1.59 (1741); *p* = .057 |
| Rumination – Reflective Pondering | | 10.99 (3.03) | 10.81 (3.26) | *t*(df) = 1.17 (1740); *p* = .243 |
| Extrinsic Social Regulation | | 11.11 (4.88) | 10.64 (4.84) | *t*(df) = 1.90 (1741); *p* = .057 |
| Extrinsic Material Regulation | | 11.39 (4.98) | 10.98 (5.11) | *t*(df) = 1.61 (1741); *p* = .107 |
| Introjected Regulation | | 19.18 (6.08) | 18.78 (6.11) | *t*(df) = 1.30 (1740); *p* = .193 |
| Identified Regulation | | 16.53 (3.99) | 16.59 (4.02) | *t*(df) = –0.28 (1741); *p* = .781 |
| Intrinsic Motivation | | 14.11 (5.15) | 14.20 (5.23) | *t*(df) = –0.34 (1741); *p* = .735 |
| Amotivation | | 6.37 (4.35) | 6.70 (4.60) | *t*(df) = –1.48 (1741); *p* = .141 |

*Note*. Statistically significant differences are shown in bold (*p* < .05).

**Gender Invariance Analysis of the BWAS**

The gender invariance of the one-factor structure of the BWAS at T1 (N = 1,473) was examined using a multigroup approach implemented in Mplus 8.10. The configural invariance model posits that the factor structure and the associations between items are equivalent for males and females, without imposing equality constraints. In the metric invariance model, all factor loadings are constrained to be equal across groups. In the strong (scalar) invariance model, both the factor loadings and item intercepts are constrained to be equal across gender groups.

Results show that the configural model demonstrated an adequate fit to the data, and the addition of constraints on factor loadings and intercepts did not result in a substantial decline in model fit, according to the recommended cutoff values of ΔCFI < .010 and ΔRMSEA < .015 (1,2). Thus, the results support configural, metric, and scalar invariance of the BWAS across gender groups (Table S3).

**Table S3**

*Results of the Gender Invariance Analysis of the BWAS (N=1473; males: 872, females: 871)*

| Model | χ^2^ (df) | AIC/BIC | RMSEA | CFI | TLI | Reference model | Δχ^2^ (p) | ΔRMSEA | ΔCFI |
| --- | --- | --- | --- | --- | --- | --- | --- | --- | --- |
| Configural invariance | 216.8 (28) | 32929.6  /33159.1 | .088 [.088; .099] | .929 | .893 |  | - | - |  |
| Metric invariance | 227.9 (34) | 32925.1 /33121.8 | .081 [.071; .091] | .927 | .910 | Configural | 7.5 (.277) | -.007 | -.002 |
| Scalar Invariance | 245.0 (40) | 32928.0  /33091.9 | .077[.068; 0.086] | .923 | .919 | Metric | 15.0 (.020) | -.004 | -.004 |

**Table S4**

*Gender Differences in Mean Scores on the Examined Variables*

|  | Males  (*N* = 285) | Females  (*N* = 301) | Difference |
| --- | --- | --- | --- |
| **Work Addiction T1** | **20.84 (5.33)** | **22.15 (4.92)** | ***t*(df) = –3.11 (584); *p* = .002** |
| **Work Addiction T2** | **18.88 (5.47)** | **20.18 (4.93)** | ***t*(df) = –3.01 (559.6); *p* = .003** |
| Self–Esteem | 29.19 (5.42) | 28.47 (5.47) | *t*(df) = 1.61 (583); *p* = .108 |
| **Self–Oriented Perfectionism** | **25.64 (6.24)** | **26.72 (6.12)** | ***t*(df) = –2.11 (582); *p* = .018** |
| Other–Oriented Perfectionism | 20.31 (5.81) | 20.48 (6.32) | *t(*df) = –0.33 (584); *p* = .741 |
| **Socially Prescribed Perfectionism** | **15.74 (6.30)** | **17.46 (7.24)** | ***t*(df) = –3.06 (584); *p* < .001** |
| Narcissism – Admiration | 10.32 (3.40) | 10.66 (3.51) | *t*(df) = –1.18 (584); *p* = .238 |
| Narcissism – Rivalry | 8.93 (3.41) | 8.59 (2.96) | *t*(df) = 1.29 (562.4); *p* = .098 |
| Depression | 12.02 (4.11) | 12.46 (4.01) | *t*(df) = –1.33 (584); *p* = .093 |
| **Anxiety** | **11.13 (3.50)** | **12.08 (3.46)** | ***t*(df) = –3.31 (583); *p* < .001** |
| **Rumination – Brooding** | **9.94 (3.17)** | **10.85 (3.16)** | ***t*(df) = –3.48 (584); *p* < .001** |
| **Rumination – Reflective Pondering** | **10.17 (3.18)** | **11.41 (3.22)** | ***t*(df) = –4.66 (584); *p* < .001** |
| Extrinsic Social Regulation | 10.29 (4.57) | 10.96 (5.07) | *t*(df) = –1.69 (582.7); *p* = .093 |
| Extrinsic Material Regulation | 11.09 (4.91) | 10.86 (5.29) | *t*(df) = 0.55 (582.8); *p* = .584 |
| **Introjected Regulation** | **17.94 (6.50)** | **19.58 (5.60)** | ***t*(df) = –3.27 (560.8); *p* = .001** |
| **Identified Regulation** | **16.02 (5.02)** | **17.13 (3.97)** | ***t*(df) = –3.35 (584); *p* < .001** |
| Intrinsic Motivation | 14.36 (5.15) | 14.05 (5.43) | *t*(df) = 0.71 (584); *p* = .238 |
| Amotivation | 6.78 (4.44) | 6.63 (4.74) | *t*(df) = 0.39 (584); *p* = .345 |

*Note*. Statistically significant differences are shown in bold (*p* < .05).

**Table S5**

*Differences in Descriptive and Work-related Variables Among the Four Groups Based on BWAS Categorization at T1 and T2.*

|  | | (1) Chronic WA group  (*N* = 103) | (2) Increased WA group  (*N* = 37) | (3) Recovered WA group  (*N* = 124) | (4) Permanently non-WA group  (*N* = 322) | Difference |
| --- | --- | --- | --- | --- | --- | --- |
| Mean age at T1 (*SD*) | | 37.66 (9.21) | 38.84 (8.50) | 36.93 (9.43) | 38.38 (9.11) | *F*(df) = 0.895 (3);  *p* = .443 |
| % of males | | 37.9 | 54.1 | 49.2 | 51.1 | *χ^2^*(df) = 6.01 (3); *p* = .111 |
| Level of education at T1  *N* (%) | less than primary level | 0 (0) | 0 (0) | 0 (0) | 1 (0.3) | χ^2^(df) = 16.91 (15); *p* = .324 |
|  | primary level | 0 (0) | 0 (0) | 1 (0.8) | 1 (0.3) |  |
|  | vocational | 0 (0) | 1 (2.7) | 1 (0.8) | 8 (2.5) |  |
|  | secondary | 22 (21.4) | 4 (10.8) | 24 (19.4) | 51 (15.9) |  |
|  | bachelor or master’s degree | 76 (73.8) | 27 (73) | 94(75.8) | 249 (77.6) |  |
|  | doctoral degree | 5 (4.9) | 5 (13.5) | 4 (3.2) | 11 (3.4) |  |
| Marital status at T1  *N* (%) | single | 54 (52.4) | 16 (43.2) | 72 (58.1) | 164 (51.1) | *χ^2^*(df) = 6.48 (9); *p* = .691 |
|  | married | 35 (34.0) | 17 (45.9) | 44 (35.5) | 125 (38.9) |  |
|  | divorced | 13 (12.6) | 4 (10.8) | 8 (6.5) | 30 (9.3) |  |
|  | widow | 1 (1) | 0 (0) | 0 (0) | 2 (0.6) |  |
| Marital status at T2  *N* (%) | single | 46 (44.7) | 9 (24.3) | 56 (45.2) | 123 (38.3) | *χ^2^*(df) = 14.02 (9); *p* = .122 |
|  | married | 42 (40.8) | 23 (62.2) | 62 (50.0) | 156 (48.6) |  |
|  | divorced | 14 (13.6) | 5 (13.5) | 6 (4.8) | 38 (11.8) |  |
|  | widow | 1 (1) | 0 (0) | 0 (0) | 4 (1.2) |  |

*Note*. Statistically significant differences (*p* < .05) are shown in bold. Superscript numbers indicate the groups that exhibit significant differences from one another.

References:

1. Cheung GW, Rensvold RB: Evaluating goodness-of-fit indexes for testing measurement invariance. Structural Equation Modeling-a Multidisciplinary Journal 2002, 9(2):233-255.

2. Dimitrov DM: Testing for Factorial Invariance in the Context of Construct Validation. Measurement and Evaluation in Counseling and Development 2010, 43(2):121-149.
